# Single-molecule observations of human small heat shock proteins in complex with aggregation-prone client proteins

**DOI:** 10.1042/BCJ20240473

**Published:** 2025-04-25

**Authors:** Lauren Rice, Nicholas Marzano, Dezerae Cox, Bailey Skewes, Antoine M. van Oijen, Heath Ecroyd

**Affiliations:** 1 Molecular Horizons and School of Chemistry and Molecular Bioscience, University of Wollongong, Wollongong, New South Wales, Australia; 2 Department of Chemistry, University of Cambridge, Cambridge, CB2 1EW, U.K

**Keywords:** αB-crystallin, Hsp27, molecular chaperones, photobleaching, proteostasis, single-molecule fluorescence

## Abstract

Small heat shock proteins (sHsps) are molecular chaperones that act to prevent the aberrant aggregation of misfolded proteins. Whilst it is suggested that sHsps prevent aggregation by binding to misfolded client proteins, the dynamic and heterogeneous nature of sHsps has hindered attempts to establish the mechanistic details of how sHsp–client protein complexes form. Single-molecule approaches have emerged as a powerful tool to investigate dynamic and heterogeneous interactions such as those that can occur between sHsps and their client proteins. Here, we use total internal reflection fluorescence microscopy to observe and characterise the complexes formed between model aggregation-prone client proteins (firefly luciferase, rhodanese and chloride intracellular channel 1 protein), and the human sHsps αB-crystallin (αB-c; HSPB5) and Hsp27 (HSPB1). We show that small (monomeric or dimeric) forms of both αB-c and Hsp27 bind to misfolded or oligomeric forms of the client proteins at early stages of aggregation, resulting in the formation of soluble sHsp–client complexes. Stoichiometric analysis of these complexes revealed that additional αB-c subunits accumulate onto pre-existing sHsp–client complexes to form larger species – this does not occur to the same extent for Hsp27. Instead, Hsp27–client interactions tend to be more transient than those of αB-c. Elucidating these mechanisms of sHsp function is crucial to our understanding of how these molecular chaperones act to inhibit protein aggregation and maintain cellular proteostasis.

## Introduction

Small heat shock proteins (sHsps) are a ubiquitously expressed, highly conserved class of intracellular molecular chaperones that are characterised by the ability to inhibit aggregation of misfolded proteins in an ATP-independent manner [1,2]. The expression of some sHsps is dramatically up-regulated in response to cellular stress, conditions conducive to protein misfolding and aggregation [3,4]. sHsps inhibit aggregation by binding to, and forming complexes with, aggregation-prone client proteins, trapping them in a state wherein they are not able to form irreversible aggregates [5,6]. Alternatively, sHsps can interact transiently with destabilised client proteins in order to briefly stabilise them and facilitate their intrinsic capacity to refold to a native state [7-9]. The dysfunction of the human sHsps HSPB1 (Hsp27) and HSPB5 (αB-c) has been implicated in a number of diseases linked to protein aggregation [10], indicating the critical role of sHsps in maintaining protein homeostasis (proteostasis). Mutations in the *HSPB1* gene encoding Hsp27 are associated with motor neuropathies such as Charcot–Marie–Tooth disease [11] and distal hereditary motor neuropathies [12-14], and overexpression of Hsp27 in an Alzheimer’s disease mouse model reduces amyloid-β plaques and improves spatial learning and memory [15]. Similarly, mutations in the *HSPB5* gene encoding αB-c [16], a major component of the eye lens, result in changes to its oligomerisation state, a reduced ability to prevent aggregation [17,18], and are causative of cataract [19].

The ability of sHsps to form polydisperse and dynamic oligomers is chiefly responsible for the absence of a mechanistic understanding regarding how these molecular chaperones prevent protein aggregation [20]. In solution, many sHsps, including αB-c and Hsp27, exist in equilibrium between large molecular mass oligomers (~18–30mers for αB-c [21-23], ~20–28mers for Hsp27 [24-26]) and smaller dissociated species (typically monomers and/or dimers). The dynamic equilibrium between sHsp oligomeric forms is impacted by post-translational modifications [24,26,27] and environmental changes [28,29], which can stimulate disassembly of the oligomers into smaller subunits that are typically thought to be the more ‘active’ chaperone forms [30-32]. Furthermore, large sHsp oligomers in complex with a client can be conformationally different from sHsp oligomers not associated with a client [33]. This highlights the difficulties in interrogating the mechanism(s) by which these heterogeneous chaperones interact with aggregation-prone proteins.

Complexes between sHsps and various clients have been previously investigated using a range of techniques, including *in vitro* light scatter assays [34], co-immunoprecipitation [34], size-exclusion chromatography (SEC) [6,30,35], and native mass spectrometry [30]. However, these previous studies have been largely limited to the characterisation of end-stage complexes since these techniques are not amenable to the interrogation of early stage sHsp–client protein complexes and how they change over time. It has been reported that, of the human sHsps (HSPB1-8), αB-c and Hsp27 are the most efficacious and promiscuous when it comes to inhibiting protein aggregation [34]. However, it remains to be resolved whether there is a shared mechanism by which these sHsps interact with client proteins and how the client protein may affect the composition of these complexes.

Single-molecule fluorescence techniques are particularly amenable to investigating dynamic and heterogeneous biological systems; thus, they are ideally suited to investigating the mechanism by which sHsps interact with client proteins [36,37]. In particular, total internal reflection fluorescence (TIRF) microscopy facilitates the determination of the stoichiometries of heterogeneous protein–protein complexes. We have recently reported the development of a single-molecule fluorescence-based technique that enabled the quantification of stable complexes formed between human αB-c and the model client protein, chloride intracellular channel 1 (CLIC), over time [38]. This approach quantifies the stoichiometries within sHsp–client complexes by using a photobleaching step-counting approach, a concept that has been widely utilised to count subunits within multi-subunit protein assemblies [37,39-41]. Here, we further exploit this technique to explore how complexes formed between human sHsps (αB-c and Hsp27) and various client proteins (firefly luciferase [FLUC], CLIC and rhodanese) evolve over time and whether there are client-dependent differences in the complexes that are formed. In doing so, we show that, once formed, the number of sHsp subunits in a sHsp–client complex increases over time. We find that substantially more αB-c subunits are recruited into sHsp–client complexes than Hsp27 subunits. Furthermore, we demonstrate that there are client-specific differences in the complexes formed between these sHsps and aggregation-prone proteins, potentially due to variations in the hydrophobicity of the client during its aggregation. Our results provide support for a common underlying mechanism by which sHsps form complexes with client proteins, whilst highlighting the heterogeneity in the sHsp–client protein complexes that are formed.

## Results

### The human sHsps αB-c and Hsp27 inhibit the aggregation of destabilised client proteins *in vitro*

Traditionally, ensemble-averaging measurements have been used to monitor the aggregation of client proteins and the ability of sHsps to inhibit this aggregation [6,33,42]. As such, we validated our investigation into the chaperone function of αB-c and Hsp27 against CLIC, FLUC and rhodanese by measuring the change in light scatter over time (indicative of amorphous aggregate formation) for each client protein, in the presence and absence of the sHsps.

**Figure 1:**
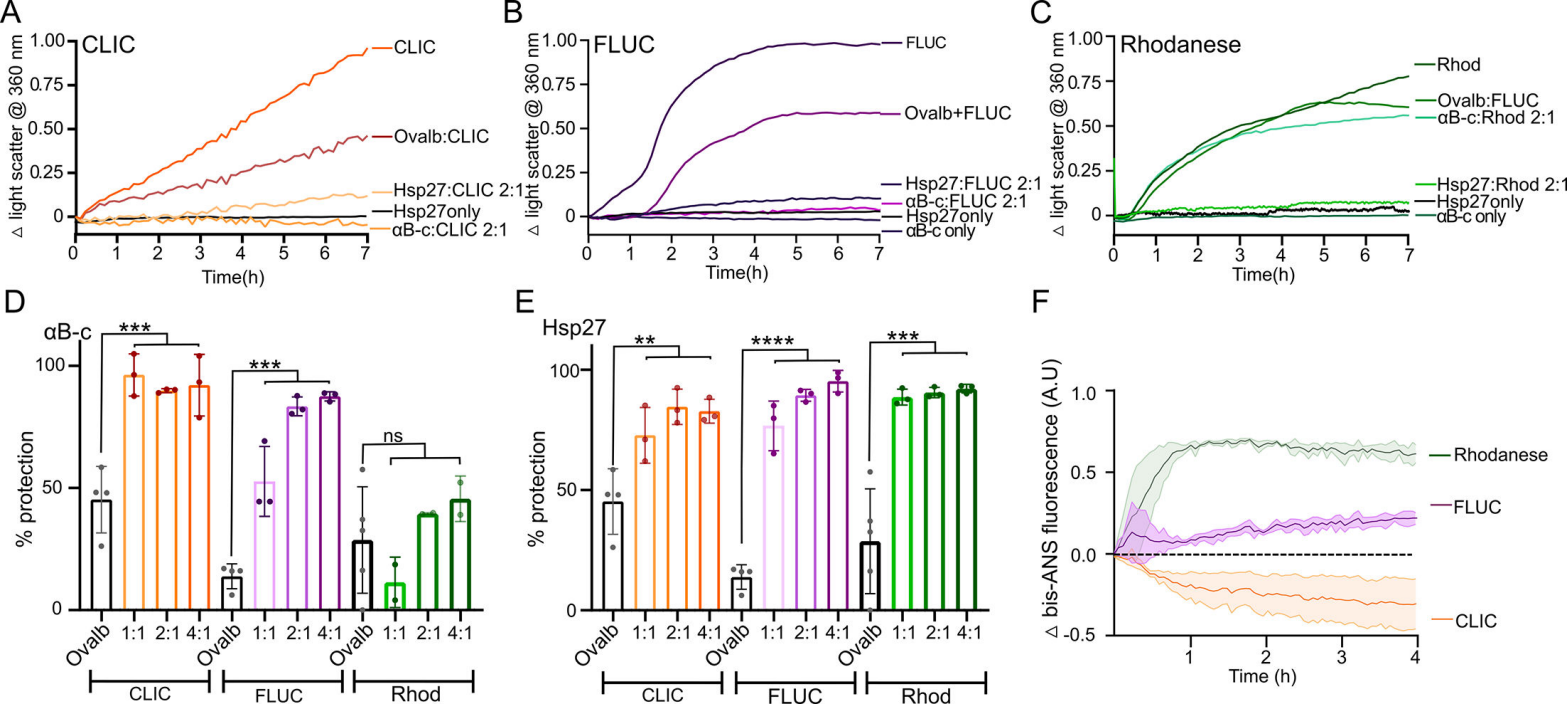
Differences in the capacity of the human sHsps, αB-c and Hsp27 to inhibit the heat-induced amorphous aggregation of client proteins. (**A-C**) Example of light scatter traces from the heat-induced aggregation of the client proteins. Recombinant client protein (A-CLIC at 30 µM, B-FLUC at 4 µM or C-rhodanese at 4 µM) was incubated at 42°C for 7 hours in the presence or absence of a 2:1 molar ratio (sHsp:client) of either αB-c or Hsp27, or the control protein ovalbumin (ovalb), and the change in light scatter at 360 nm over time monitored. Data shown are the normalised change in light scatter. (**D-E**) The percent reduction in aggregation (light scatter) of CLIC, FLUC and rhodanese afforded by varying molar ratios of αB-c (**D**) and Hsp27 (**E**), and the control protein ovalbumin at the highest molar ratio tested (4:1, ovalb:client). Data reported are the average ± S.D. of three independent experiments. Data were analysed by one-way ANOVA and Tukey’s post-hoc test, where ****, *** and ** indicates *P*<0.0001, 0.001 and 0.01, respectively; ns indicates *P*>0.05. (**F**) Exposed hydrophobicity of client proteins was monitored by bis-ANS fluorescence. CLIC, FLUC and rhodanese (200 nM) were incubated at 42°C for 4 hours in the presence of 20 µM bis-ANS; the change in fluorescence emission from bis-ANS at 480 nm was monitored over time. Data are reported as the mean ± S.E. of three independent experiments. CLIC, chloride intracellular channel 1; FLUC, firefly luciferase; sHsps, small heat shock proteins.

When each client was incubated in the absence of sHsps, an increase in light scatter over time was observed, indicating that aggregation had occurred (Figure 1A–C). However, the time taken for each client to aggregate varied substantially, despite them being subjected to identical incubation conditions (Figure 1A–C). The light scatter associated with the aggregation of CLIC did not reach a plateau even after 20 hours of incubation (Figure 1A; Supplementary Figure S1), whilst for FLUC and rhodanese, the amount of light scatter either reached or approached a plateau after only 7 hours, respectively (Figure 1B–C). When either CLIC or FLUC was incubated in the presence of αB-c, there was a significant decrease in light scatter (*P*<0.005), consistent with the inhibition of protein aggregation (Figure 1D). Notably, αB-c did not significantly inhibit the aggregation of rhodanese – the decrease in light scatter was comparable with the control protein, ovalbumin. When all three clients were incubated in the presence of Hsp27, there was a significant reduction in light scatter consistent with the inhibition of aggregation (Figure 1E). Whilst the presence of ovalbumin did lead to a decrease in light scatter for all three client proteins tested, this did not occur to the same extent as for the sHsps – with the exception of the αB-c and rhodanese pair noted previously – and is consistent with previous studies that have shown that albumin demonstrates weak chaperone-like activity against some clients [43,44].

We next investigated the amount of exposed hydrophobicity on the client protein during heat-induced unfolding; to do so, we performed 1,1′-bi(4-anilino)naphthalene-5,5′-disulfonic acid (bis-ANS) assays (Figure 1F). When FLUC and rhodanese were incubated with bis-ANS under the same conditions used for the aggregation assays (Figure 1A–C), there was an increase in bis-ANS fluorescence over time that is indicative of an increase in surface-exposed hydrophobicity upon client misfolding. However, the increase in fluorescence occurred faster and to a greater extent for rhodanese compared with FLUC. In contrast, bis-ANS fluorescence decreased upon incubation with CLIC, which suggests that CLIC misfolding results in hydrophobic regions becoming more buried when compared with the native state.

Collectively, these data confirm that sHsps inhibit the overall aggregation of these client proteins and that the extent to which this occurs varies depending on the client. Differences in the capacity of αB-c and Hsp27 to inhibit the aggregation of the various clients could relate to varying degrees of surface-exposed hydrophobicity during the early stages of client misfolding. However, specific mechanistic differences that may underlie these observations were not able to be determined using these techniques as they measure the average of all molecules in solution; as such, we turned to a single-molecule approach to further interrogate these sHsp–client protein interactions.

### The number of αB-c subunits in complex with client proteins increases over time and is client-dependent

**Figure 2:**
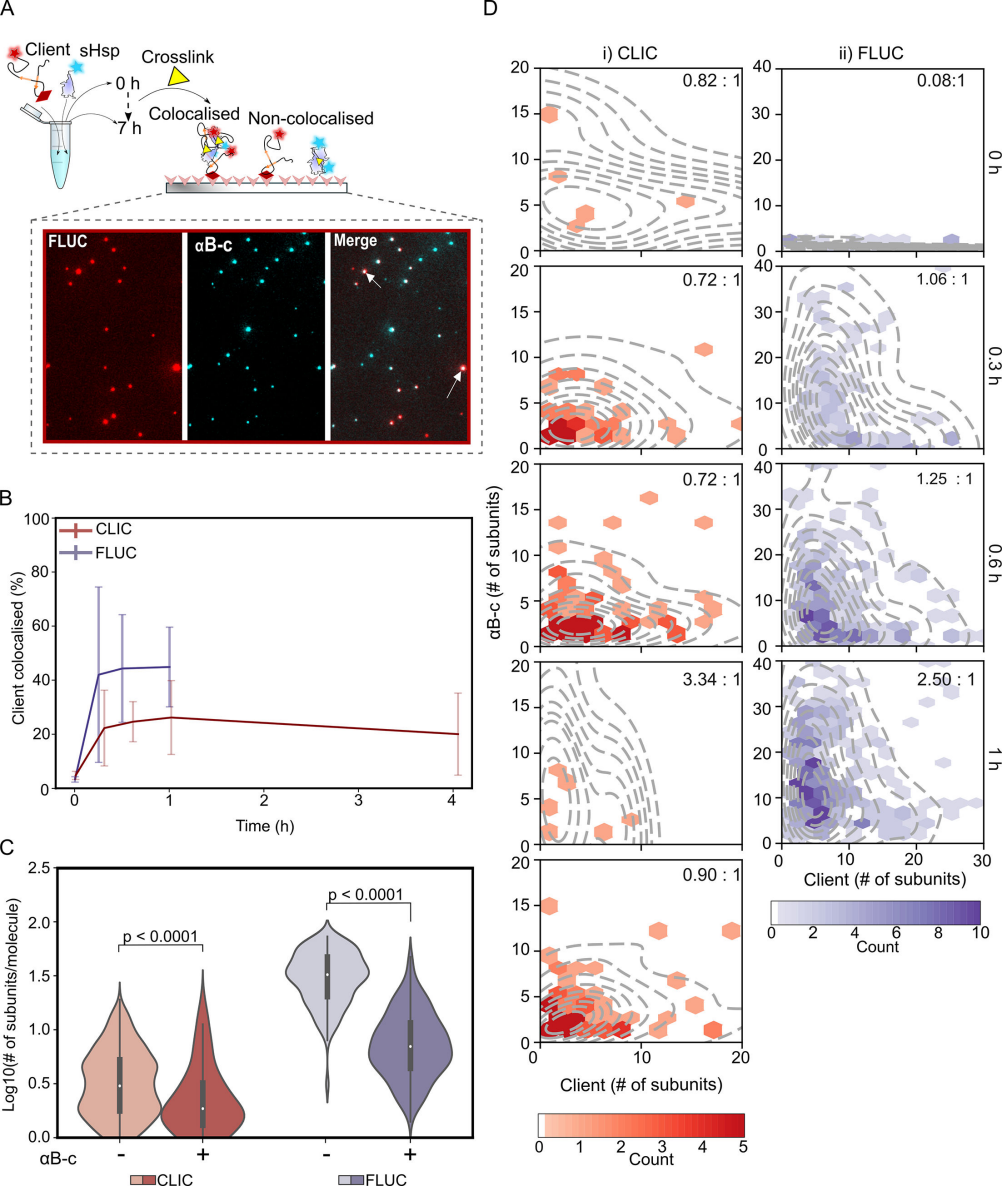
The number of αB-c subunits within sHsp–client protein complexes increases over time in a client-dependent manner. αB-c-AF488 and CLIC- or FLUC- AF647 were incubated (42°C for up to 7 hour) in the absence or presence of αB-c (2:1 molar ratio; αB-c:client); aliquots were taken at time points throughout the incubation. Following incubation, samples were immediately cross-linked, diluted and incubated in flow cells for 15 min before imaging using TIRF microscopy. (**A**) Schematic depicting the workflow used to identify sHsp–client complexes formed during incubation. Representative TIRF microscopy image shows the fluorescence emission from FLUC-AF647 and αB-c-AF488 when incubated together, observed in separate emission channels. Overlaying these channels (‘Merge’) allows identification of colocalised (white arrows) and non-colocalised molecules. (**B**) The percentage (%) of CLIC-AF647 (red) or FLUC-AF647 (purple) molecules colocalised with αB-c-AF488 at each time point, reported as the mean ± S.D. of three independent replicates. (**C**) Violin plots show the distributions of CLIC-AF647 (red) and FLUC-AF647 (purple) molecule sizes (log_10_ number of subunits/molecule) after 1 hour (for FLUC) or 4 hours (for CLIC) incubation with (‘+’) or without (‘-’) αB-c-AF488. Molecule size reported includes all molecules (i.e. both colocalised and non-colocalised with αB-c). Results include measurements from three independent experiments and, where marked, statistical comparisons between distributions was performed via Kruskal-Wallis test for multiple comparisons with Dunn’s procedure (p values indicated). (**D**) Hexbin plots show the relative abundance of protein subunits and median molar ratio (sHsp:client) (inset) of αB-c-AF488 (y-axis) and CLIC-AF647 (i) or FLUC-AF647 (ii) (x-axis) within complexes at each time point during incubation. Each hexbin plot is overlaid with the kernel probability density (dashed line) of complexes at each time point. The depth of colour in each hexbin indicates the number of molecules in that bin, the scale for each plot is indicated by the respective colour bar. Data shown are from all molecules in complexes measured across the three independent experiments. CLIC, chloride intracellular channel 1; FLUC, firefly luciferase; sHsps, small heat shock proteins.

To interrogate the interaction between the sHsps αB-c and Hsp27 with these model client proteins, we utilised a previously described single-molecule approach that enables the identification and characterisation of sHsp–client complexes [38]. Briefly, this approach involves incubation of Alexa Fluor 647 (AF647)-labelled client proteins in heat-denaturing conditions in the absence or presence of Alexa Fluor 488 (AF488)-labelled sHsps. Aliquots are taken over the course of the reaction (Figure 2A), cross-linked to prevent complex dissociation (Supplementary Figure S2), diluted and immobilised on a coverslip for TIRF microscopy imaging until all fluorophores are photobleached. The spectrally distinct fluorophores attached to the client or sHsp are visible in separate imaging channels which, when aligned and overlaid, enable identification of coincident fluorescent spots indicative of sHsp–client complexes (Figure 2A). We used this approach to investigate the sHsp–client combinations in which client protein aggregation was significantly inhibited by the sHsp in the ensemble-averaged assays, that is, αB-c with either FLUC or CLIC; Hsp27 with all three clients (Figure 1).

Within 0.3 hours of incubation, ~20% of immobilised CLIC molecules were colocalised with αB-c, indicating that only a small proportion of sHsp–client complexes formed in this time, and this proportion of colocalisation remained consistent throughout the remainder of the time course (Figure 2B). This is consistent with our previous observations of this sHsp–client pair that did not involve cross-linking of complexes [38], indicating that the chemical cross-linker does not alter complex formation in this case. When FLUC was incubated with αB-c under the same conditions, ~40% of FLUC molecules were colocalised with αB-c within 0.3 hours (Figure 2B). The increased formation of colocalised complexes between αB-c and FLUC compared with CLIC is consistent with sHsps preferentially interacting with clients that have increased hydrophobicity and aggregation propensity (Figure 1F). However, despite there being a two-fold molar excess of chaperone in these assays, 100% colocalisation of FLUC molecules with αB-c was not observed, suggesting that sHsps selectively form complexes with a sub-population of client molecules. This sub-population may be those that are more aggregation-prone. Importantly, negligible colocalisation (<3%) was observed when an AF647-labelled non-chaperone protein (single-stranded binding protein, SSB) and the AF488-labelled client protein (rhodanese) were incubated under the same conditions as for each sHsp–client protein pair (Supplementary Figure 3 ). This confirms that colocalisation between sHsps and a client are the result of specific interactions between the proteins, rather than non-specific interactions between proteins or the attached fluorophores. Moreover, the incubation of fluorescently labelled sHsps and clients often resulted in observable fluorescence resonance energy transfer (FRET) within sHsp–client complexes, which was not present when the client was incubated with the non-chaperone protein SSB (Supplementary Figure 4 ). This serves as further evidence that the colocalisation of sHsps and clients observed in these experiments are the result of sHsp–client complexes that are formed.

Using this single-molecule approach, it was also possible to determine the size (i.e. number of subunits per client molecule) of each client species when incubated in the presence or absence of αB-c. When CLIC or FLUC were incubated with αB-c, the client species observed were significantly smaller than those incubated in the absence of αB-c (Figure 2C, *P*<0.0001), demonstrating that the formation of complexes acts to inhibit aggregation of both clients. As expected, both client species were smallest prior to incubation, indicating that any increase in size is indeed due to aggregation (Supplementary Figure S5). Interestingly, when incubated with αB-c, the distribution of client molecule sizes was broader than those seen prior to incubation (Supplementary Figure S5). This indicates that some client molecules were still able to increase in size despite a proportion of them forming complexes with αB-c.

We next determined the composition of the sHsp–client complexes by calculating the ratio of client to sHsp molecules within individual complexes over time. When αB-c was in complex with CLIC, the relative molar ratio of αB-c:CLIC fluctuated from 0.72:1 to 3.34:1 over time (Figure 2D–i), although this change was not statistically significant (*P*=0.24), which is mostly attributable to the very few αB-c-CLIC complexes able to be observed at each time point. This suggests that, given the surface-exposed hydrophobicity of CLIC decreases under these incubation conditions (Figure 1F), formation of complexes between αB-c and CLIC is not favoured. In contrast, the distribution of αB-c:FLUC molar ratios within complexes increased significantly over time (*P*<0.0001) – the median increased from 0.08:1 (prior to incubation, i.e. 0 hours) to 2.5:1 after 1 hour (Figure 2D–ii). FLUC species in complex with αB-c remained significantly smaller than FLUC species incubated alone (Figure 2C). Interestingly, neither αB-c-FLUC complexes nor FLUC molecules not in complex with αB-c, were able to be observed after 4 hours of incubation. In line with previous work regarding the mechanism of action of αB-c [38], these data suggest that αB-c initially forms stable complexes with FLUC and then additional αB-c subunits are recruited into these complexes over time; in doing so, extensive aggregation of FLUC is inhibited (Figure 2D). The subsequent decrease in the total number of αB-c-FLUC complexes able to be observed at later time points suggests that, within the largest complexes, the manner by which the αB-c subunits associate with the FLUC molecules prevents them from binding to the coverslip. Taken together, these data show that αB-c forms stable complexes with both CLIC and FLUC. Differences in the extent and manner by which these complexes are formed are likely due to differences in hydrophobicity between the two clients. To determine whether these findings are unique to αB-c or are a more general mechanism by which human sHsps interact with client proteins, similar experiments were performed with Hsp27.

### Hsp27 prevents client aggregation through complex formation and transient interactions

**Figure 3:**
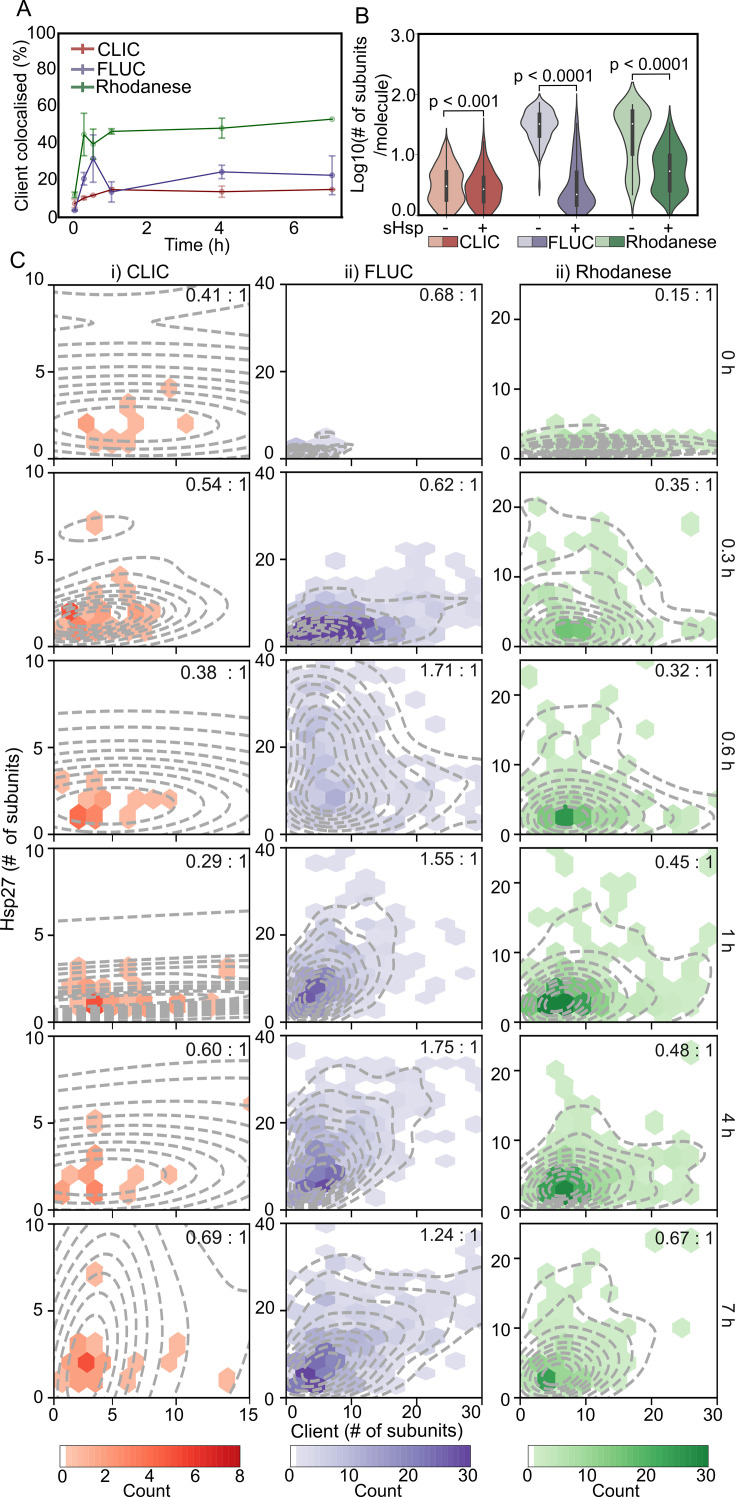
The number of subunits of Hsp27 within complexes increases in a client-dependent manner. Hsp27-AF488 and the client proteins (CLIC-, FLUC- or rhodanese-AF647) were incubated (42°C for up to 7 hour) in the absence or presence of Hsp27 (2:1 molar ratio, Hsp27:client); aliquots were taken at time points throughout the incubation. Following incubation, samples were immediately cross-linked, diluted and incubated in flow cells for 15 min before imaging using TIRF microscopy. (**A**) The percentage (%) of CLIC-AF647 (red), FLUC-AF647 (purple) or rhodanese-AF647 (green) molecules colocalised with Hsp27-AF488 at each time point, reported as the mean ± S.D. of three independent replicates . (**B**) Violin plots show the distributions of CLIC-AF647 (red), FLUC-AF647 (purple) and rhodanese-AF647 (green) molecule sizes (log_10_ number of subunits/molecule) after 7-hour incubation with (‘+’) or without (‘-’) Hsp27-AF488. Molecule size reported includes all molecules (i.e. both colocalised and non-colocalised with Hsp27). Results include measurements from three independent experiments and, where marked, statistical comparisons between distributions was performed via Kruskal-Wallis test for multiple comparisons with Dunn’s procedure (p values indicated). (**C**) Hexbin plots show the relative abundance of protein subunits and median molar ratio (sHsp:client) (inset) of Hsp27-AF488 (y-axis) and each of the clients (CLIC-AF647 (i), FLUC-AF647 (ii) and rhodanese-AF647 (iii)) (x-axis) within complexes at each time point during incubation. Each hexbin plot is overlaid with the kernel probability density (dashed line) of complexes at each time point. The depth of colour in each hexbin indicates the number of molecules in that bin, the scale for each plot is indicated by the respective colour bar. Data shown are from all molecules in complexes measured across the three independent experiments. CLIC, chloride intracellular channel 1; FLUC, firefly luciferase; sHsps, small heat shock proteins.

We sought to determine whether the interaction between Hsp27 and the model client proteins CLIC, FLUC and rhodanese was comparable to αB-c. As for αB-c, the percentage of CLIC molecules that colocalised with Hsp27 increased in the first hour to 15% (Figure 3A). Notably, Hsp27 more rapidly colocalised with rhodanese compared with CLIC, such that ~40% of rhodanese molecules were colocalised with Hsp27 within 0.3 hours of incubation (Figure 3A). Colocalisation of Hsp27 with both rhodanese and CLIC plateaued after 1 hour (Figure 3A). When Hsp27 was incubated with FLUC, the proportion of FLUC molecules within complexes was dynamic; within the first 0.6 hours of incubation, there was a rapid increase in colocalisation to ~35% and this was followed by a two-fold decrease in colocalisation (to ~17%) after 1 hour. Unlike αB-c (Figure 2), there was no decrease in the number of observed FLUC molecules bound to the coverslip surface after extended incubation. Collectively, these results show that despite Hsp27 inhibiting the aggregation of all clients (Figure 1), colocalisation with the client molecules varies over time and between clients. As such, there may be differences in the mechanism by which Hsp27 forms complexes with different clients.

To interrogate this, we first determined the effect of Hsp27 on the molecule size of each client species regardless of whether it was colocalised with Hsp27 or not (Figure 3B). Consistent with results observed for αB-c, the size distribution of all three clients was significantly smaller when incubated in the presence of Hsp27 (*P*<0.0001). Again, as expected, the FLUC and rhodanese molecules were small prior to incubation (Supplementary Figure S6), and thus, the increase in size in the absence of Hsp27 was attributable to aggregation. Additionally, the distribution of CLIC and FLUC molecule sizes when incubated with Hsp27 was broader than those seen prior to incubation (Supplementary Figure S6).

The stoichiometric distribution of complexes between CLIC and Hsp27 ranged from a median Hsp27:CLIC ratio of 0.29:1 to 0.69:1 (Figure 3C(i)); however, as with the αB-c and CLIC pair, there were very few observable Hsp27-CLIC complexes. There were differences in the molar ratio of the Hsp27–client complexes that formed over time between rhodanese and FLUC (Figure 3E(ii,iii)). Whilst only smaller species of Hsp27 (i.e. monomers or dimers) were present in complexes with FLUC at early time points, the number of Hsp27 molecules in complexes increased ~2.5 fold within 4 hours of incubation (from 0.68:1 to 1.75:1, *P*<0.0001) and then decreased after 7 hours to 1.24:1 (*P*<0.05). Importantly, even at the maximum average molar ratio of Hsp27:FLUC, the number of Hsp27 subunits within complexes was still only half that of αB-c under the same conditions (see Figure 2). Prior to incubation (0 hours), monomers and dimers of Hsp27 bound to a distribution of rhodanese oligomers (1–20 subunits, median molar ratio of 0.15:1). The molar ratio of Hsp27–rhodanese complexes increased steadily throughout the incubation, reaching a maximum of 0.67:1 after 7 hours, indicating recruitment of Hsp27 molecules into complexes with rhodanese over time. However, these complexes always contained less Hsp27 subunits per rhodanese molecule compared with those complexes formed with FLUC. These data show that, similar to αB-c, Hsp27 binds CLIC, FLUC and rhodanese over time to different extents and, in doing so, prevents the formation of large client aggregates. In contrast with αB-c, Hsp27 displayed dynamic complex formation with FLUC and the complexes that formed comprised less sHsp molecules per FLUC monomer.

### αB-c maintains client proteins in small oligomeric states

**Figure 4:**
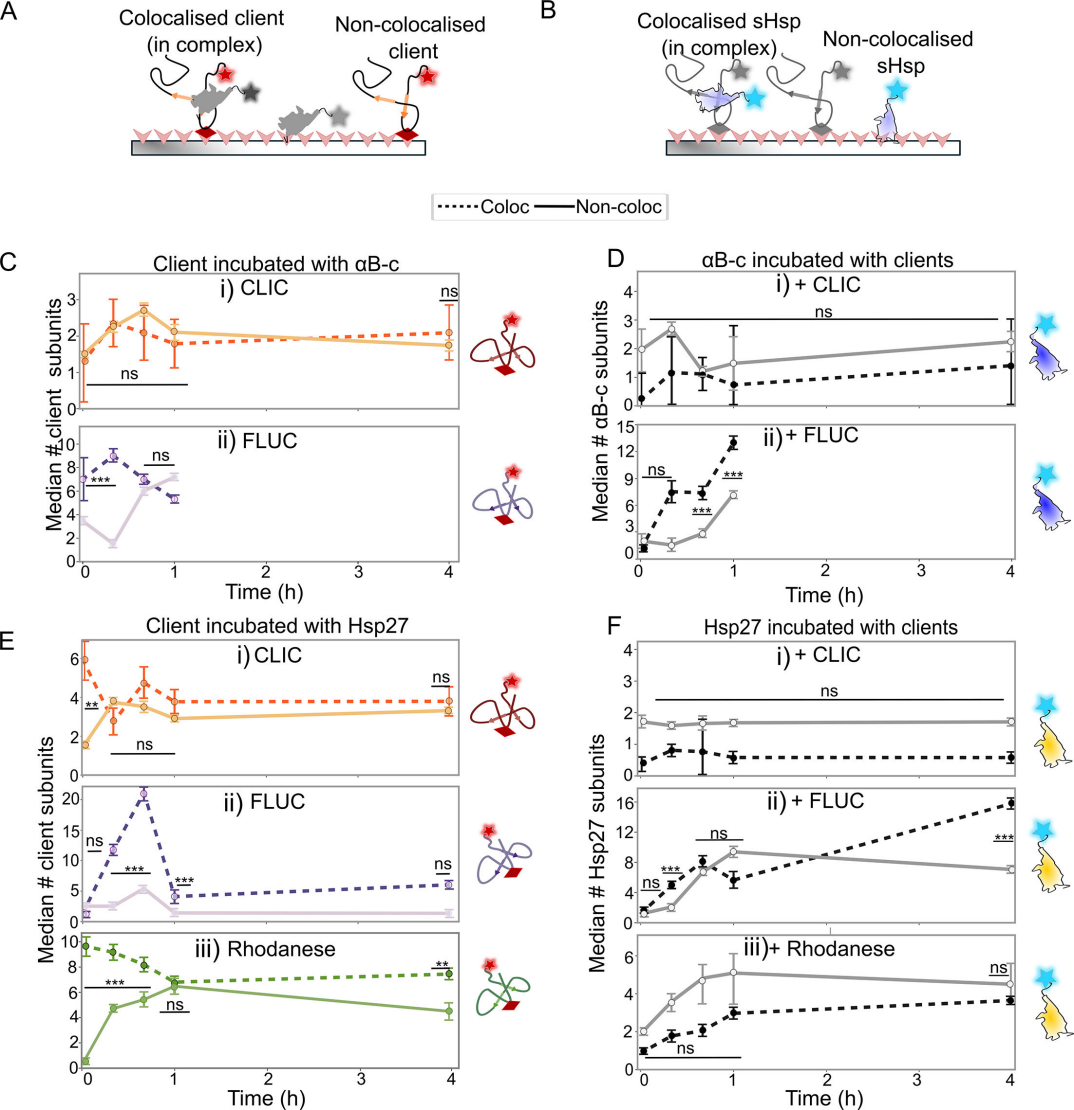
sHsps maintain client proteins in small oligomeric states. AF488-labelled sHsps (αB-c or Hsp27) were incubated with the AF647-labelled client proteins (CLIC, FLUC or rhodanese) (42°C for 4 hour, 2:1 molar ratio) and aliquots were taken throughout the incubation. Samples were immediately cross-linked, diluted and incubated in flow cells for 15 min before imaging using TIRF microscopy. The molecule size (# of subunits/molecule) was calculated for all molecules, and the data were filtered for the (**A**) client and (**B**) sHsp molecules that were colocalised with one another (i.e. in complexes) and those that were not colocalised (i.e. not in complexes). (**C-E**) The median (±S.E.) number of subunits per molecule of both colocalised (‘Coloc’) and non-colocalised (‘Non-coloc’) molecules was plotted at each time point for the clients CLIC (i), FLUC (ii) or rhodanese (E-iii) incubated with (**C**) αB-c or (**E**) Hsp27. The size of the sHsps in these treatments are shown in the corresponding plots for (**D**) αB-c and (**F**) Hsp27. The median is calculated at each time point from all molecules from three independent experiments. Comparisons were made via a two-way ANOVA with Tukey’s HSD post-hoc testing performed to compare the number of subunits between colocalisation states (i.e. ‘Coloc’ versus ‘Non-coloc’) at each time point; ns = not significant (*P*>0.05); *** *P*<0.0005; **P<0.005. CLIC, chloride intracellular channel 1; FLUC, firefly luciferase; sHsps, small heat shock proteins.

To better understand how the sHsps recognise and maintain the entire population of client protein molecules in a non-aggregated state despite not forming complexes with all client molecules (Figures 2B and 3A), we directly compared the size of the non-colocalised client molecules (i.e. not in complexes with a sHsp) at each time point with those that were colocalised (i.e. in complexes) (Figure 4A and C). The number of CLIC subunits in complex with αB-c was similar to those not in complex throughout the incubation period (*P*>0.05) (Figure 4C(i)). In contrast, at early time points (i.e. <1 hour), the number of FLUC subunits per molecule was significantly higher when in complex with αB-c compared with those not in complex (*P*<0.01) (Figure 4C(ii)), suggesting that αB-c selectively binds small oligomeric FLUC species rather than native or misfolded monomers. However, after 1 hour of incubation, the size of the FLUC molecules not in complexes significantly increased to ~8 subunits (*P*<0.01, Supplementary Figure S7A(ii)), while the number of FLUC subunits in complexes remained similar throughout the incubation (~8 subunits, *P*>0.05, Supplementary Figure S7A(ii)). The size of CLIC molecules not in complexes significantly increased during the first hour of incubation, before decreasing to be the same size as those present prior to incubation (Supplementary Figure S7A(i), *P*<0.05), which suggests that initial associations between CLIC molecules are not stable.

Next, we determined whether the change in the size of each of the sHsps over time differed between those that were in complexes compared with those that were not. When we compared the number of αB-c subunits per molecule when incubated with CLIC (Figure 4D(i)), we observed that small αB-c oligomers initially bind to CLIC. The size of the αB-c bound to CLIC did not increase over time (Supplementary Figure S7B(i), *P*>0.05) and remained similar to non-colocalised αB-c (Figure 4D–i, *P*>0.05). When incubated with FLUC, the number of colocalised αB-c subunits per complex increased rapidly in the first hour (~13 subunits/complex after 1 hour) (Figure 4D–ii; Supplementary Figure S7B(ii), *P*<0.05). Interestingly, the size of non-colocalised αB-c also increased between 0.6 and 1 hour; however, this occurred at a slower rate and to a lesser extent (~6 subunits/oligomer after 1 hour) compared with the colocalised αB-c (Supplementary Figure S7B(ii), *P*<0.05). These data suggest that αB-c binds to FLUC molecules that have already begun to aggregate, preventing their further aggregation; over time, small αB-c subunits are recruited into complexes such that, at later time points, there are fewer subunits available to inhibit the aggregation of FLUC. Since this work has demonstrated that the smaller sHsp species (i.e. monomers/dimers) preferentially associate with the misfolded protein, it is likely that these are depleted from solution first and sequestered into sHsp–client complexes. This would account for the observed increase in the median size of non-colocalised αB-c (Figure 4D(ii)).

### Hsp27 prevents aggregation through stable and transient interactions

Next, we compared the number of CLIC, FLUC and rhodanese subunits within complexes formed with Hsp27 with those not in complexes (Figure 4E(i-iii)). The number of CLIC (Figure 4E(i)) and rhodanese (Figure 4E(iii)) subunits per molecule was initially higher when in complex with Hsp27 compared with those not in complex with Hsp27 (0–0.6 hours, *P*<0.05). However, the median number of rhodanese molecules within these complexes decreased at later time points (>1 hour, Supplementary Figure S7C(iii), *P*<0.05), whilst the number of CLIC molecules within complexes with Hsp27 did not change (Supplementary Figure S7C(i); *P*>0.05). Conversely, the median size of the client molecules (both CLIC and rhodanese) not in complex with Hsp27 increased over time (Supplementary Figure S7C(i) and (iii)), such that they were approximately the same size as those molecules in complex (Figure 4E(i) and (iii); *P*>0.05). This supports the hypothesis that for these clients, molecules that are not initially bound by a sHsp eventually begin to oligomerise and aggregate.

Prior to incubation (0 hours), the FLUC molecules that were both in and out of complexes with Hsp27 were predominantly present as small species (i.e. 2 subunits) (Figure 4E(ii); *P*>0.05). However, the median number of FLUC subunits in complexes with Hsp27 increased significantly until 0.6 hours, reaching up to 20 subunits per complex (Supplementary Figure S7C(ii), *P*<0.001). By 1 hour, the number of FLUC subunits in complexes decreased significantly compared with the prior time points (Supplementary Figure S7C(ii), *P*<0.05), becoming similar in size to non-colocalised FLUC molecules (~5 FLUC subunits/complex) (Figure 4E(ii)). The FLUC molecules that were not in complexes also increased in size in the first 0.6 hours of incubation (Supplementary Figure S7C(ii); *P*<0.05), before decreasing in size at each remaining time point (Supplementary Figure S7C(ii), *P*<0.05). However, the magnitude of these changes was smaller than for those that were in complex with Hsp27. Thus, throughout the incubation, the FLUC molecules that were not in complex with Hsp27 remained smaller (<5 subunits per FLUC molecule) than those that were in complex with Hsp27 (Figure 4E(iii), *P*<0.05). This is in stark contrast to results observed in the presence of αB-c, whereby the non-colocalised FLUC increased significantly in size. Taken together, these data suggest that Hsp27 inhibits aggregation through both stable and transient interactions with aggregation-prone forms of client proteins.

Finally, we compared the number of Hsp27 subunits per molecule when non-colocalised or in complex with clients. Prior to incubation, Hsp27 molecules, whether in complex with client proteins or not, were monomers and dimers (Figure 4F(i,iii); *P*>0.05). When Hsp27 was incubated in the presence of CLIC (the least aggregation-prone client with the lowest propensity to form complexes), the number of Hsp27 subunits per molecule, both for those within complexes and not, did not change over time (1–2 subunits/molecule) (Supplementary Figure S7D(i); *P*>0.05). In contrast, when incubated in the presence of FLUC or rhodanese, the number of Hsp27 subunits per molecule increased throughout the incubation period (Supplementary Figure S7D(ii) and (iii), *P*<0.05). For rhodanese, the increase in the number of Hsp27 molecules was more gradual for those in complexes and resulted in there being no difference between the number of Hsp27 subunits in molecules in or out of complexes (Figure 4F(iii); *P*>0.05). This trend was similar for Hsp27 and FLUC, except for at 4 hours, at which point there were more Hsp27 subunits in complexes with FLUC than when not in complex (Figure 4F(ii); *P*<0.05). Similar to αB-c, this could be due to the smaller monomeric and/or dimeric species of Hsp27 being depleted from solution as they bind the client.

## Discussion

It is well known that the sHsps αB-c and Hsp27 form a range of oligomers in the absence of a client and that when a client is present, they can form large, polydisperse sHsp–client complexes [2,30,34,45]. However, it remains to be resolved whether the mechanism by which these sHsps form complexes with clients is shared, and whether the formation of complexes is influenced by the client protein present. This study reports on the heterogeneous complexes that form between sHsps and aggregation-prone client proteins, revealing a common underlying mechanism by which sHsps interact with these client proteins to prevent aggregation.

### αB-c and Hsp27 share a common mechanism to inhibit client protein aggregation

Our data suggest that, at early time points, αB-c and Hsp27 behave similarly to inhibit aggregation of client proteins. The application of heat induces the client protein to misfold, which in some cases results in exposure of hydrophobic regions on the polypeptide, and the formation of small oligomeric/early aggregated client species. It is well known that, when exposed to external stressors such as heat, polypeptides can adopt a variety of misfolded conformations [46] and that sHsps preferentially interact with regions of high hydrophobicity on client proteins [2,7,8,47-49]. As such, the affinity of an individual sHsp subunit for the hydrophobic client may become significantly higher than its affinity for other sHsp subunits or less hydrophobic conformations of client proteins. This would result in a decrease in the size of the larger sHsp oligomers [50,51], increased availability of free sHsp monomers and dimers in solution and would contribute to the rapid formation of early sHsp–client complexes with aggregation-prone client molecules. This is consistent with our observations that complex formation for both αB-c and Hsp27 occurs within the first 0.3–1 hours following incubation with a client (Figures 2B and 3A). Moreover, complex formation – and the extent to which this occurs – is enhanced for clients for which there is a significant increase in hydrophobicity during this same time period (i.e. rhodanese, Figure 1F), and as such, these clients may require rapid, stable complex formation by sHsps to inhibit aggregation [52,53]. This is also apparent when comparing the early complex formation of Hsp27 with FLUC and rhodanese; Hsp27 forms a greater proportion of stable complexes with rhodanese than it does FLUC, which correlates with the differences in hydrophobicity between these two clients at this time (i.e. rhodanese hydrophobicity increased to a level ten times higher than that of FLUC in the first 1 hour).

The results from this work indicate that, after the initial periods during which sHsp–client complexes form (i.e. up to 1 hour), additional sHsp subunits are recruited from solution into the complexes, resulting in larger complexes with more sHsps bound. The ‘seeding’ or additional recruitment of these sHsp subunits into larger complexes has been suggested previously as a mechanism by which αB-c inhibits aggregation [38]. Here, we demonstrate that this is a common underlying mechanism shared by both αB-c and Hsp27. This mechanism is also consistent with previous work which proposed that two distinct populations of sHsp-client protein complexes exist – a less abundant population that forms the inner-core of the complex with the client, and a more abundant surface-exposed and dynamic outer shell population [54]. Our data suggest that the dynamic outer shell population consists of those sHsp subunits recruited from solution into complexes once the stable early stage sHsp–client complexes have formed.

Whilst the number of αB-c and Hsp27 subunits in complexes increases over time, the number of client molecules in the complexes remains relatively stable, leading to an increase in the sHsp:client molar ratio of the complexes. The increase in the number of sHsp subunits in complexes over time is particularly apparent for those client proteins which form larger aggregates in the absence of a sHsp (i.e. FLUC and rhodanese). Interestingly, complexes formed with FLUC (the largest client protein, at 62 kDa) had the most sHsp subunits compared with complexes formed with CLIC (27 kDa) or rhodanese (35 kDa). Recruitment of additional sHsp subunits from solution into existing complexes probably occurs by a combination of sHsp self-oligomerisation via the disordered C- and N-terminal regions [2], as well as the binding of sHsps to binding sites on the client polypeptide that become exposed over time [46]. The extent to which this recruitment occurs may relate to the molecular mass of the client protein, since the availability of binding sites on a larger client may be greater. Evidence from this work to support this is that the complexes Hsp27 formed with FLUC (the largest client protein, at 62 kDa) have the largest number of sHsp subunits compared with complexes formed with CLIC (27 kDa) or rhodanese (35 kDa) (Supplementary Figure S8).

### Differences in the complexes formed by αB-c and Hsp27 with client proteins

Differences in the complexes formed between αB-c and Hsp27 with FLUC became most apparent at later time points during incubation (from 1 hour onwards), with no αB-c-FLUC complexes detected with our single-molecule approach after 4 hours. Since we observe substantial recruitment of αB-c subunits to complexes over time, we surmise that the recruited αB-c within these complexes shields the C-terminal biotin moiety on FLUC such that it can no longer bind to the NeutrAvidin functionalised coverslip. In contrast, the relative proportion of FLUC molecules within Hsp27-FLUC complexes was found to decrease after 1 hour of incubation, suggesting that some of the Hsp27-FLUC complexes dissociate, resulting in more non-colocalised FLUC subunits. Of note, despite some Hsp27-FLUC complexes dissociating, many large Hsp27-FLUC complexes remained at the end of the incubation (in contrast to αB-c), suggesting that the structural interactions between Hsp27 and FLUC in these complexes are dynamic and heterogeneous.

The difference in surface-binding capacity between the complexes formed between FLUC and either Hsp27 or αB-c indicates that, as for other techniques dependent on protein ‘capture’ (e.g. surface plasmon resonance, co-immunoprecipitation), burial of the biotin tag within large sHsp–client complexes can prevent their ability to be captured, detected and characterised by this TIRF-based single-molecule assay. Despite this limitation, this technique is uniquely suited for the detection and characterisation of early stage complexes that form between sHsps and aggregation-prone client proteins. Traditionally, approaches such as SEC, electron microscopy and native mass spectrometry have been employed to study end-stage complexes formed between sHsps and their clients. However, they are not capable of providing the level of detail of individual complexes in a heterogeneous sample as can be achieved with our single-molecule approach. More work is required to understand the interactions within complexes that may influence surface immobilisation in this assay and this could incorporate complementary solution-based techniques such as single-molecule confocal microscopy, which can report on the distribution of all molecules in solution.

A limitation of our single-molecule approach is the need to cross-link samples to facilitate their characterisation at the single-molecule level. Cross-linking ensures that any complexes – stable or transient – present in solution at the time are captured and therefore represented in the data; however, it is acknowledged that this can distort the equilibrium, in particular by stabilising (and hence enhancing) transient interactions that are present. Nonetheless, we were able to show via our single-molecule technique that whilst aggregation (as detected by light scatter) is inhibited overall in the presence of the sHsps, there is a proportion of client molecules that do not form complexes with sHsps and these can increase in size during incubation. For example, when FLUC and αB-c, or rhodanese and Hsp27, were incubated together, the non-colocalised client molecules increased in size over time, suggesting incomplete inhibition of aggregation. However, when FLUC was incubated with Hsp27, those molecules not in complex remained small throughout the entire incubation.

Taken together, these data suggest that the formation of stable complexes with clients is not the sole mechanism through which sHsps inhibit aggregation. Transient interactions, such as those that take place between Hsp27 and FLUC, may also take place between sHsps and other client proteins and these too can act to inhibit the client from forming aggregates. Substrate- and conformation-specific interactions have been previously observed for αB-c [8] as well as the sHsps HSPB4 and HSPB8 [48,49], including the capacity to interact transiently with some aggregation-prone states of client proteins, whilst forming stable complexes with others. Thus, client protein molecules that are not within stable complexes but do not aggregate, may be those misfolded conformations which are only involved in low-affinity, transient interactions with sHsps.

It has been previously suggested that the interaction of sHsps, including Hsp27, with FLUC may act to facilitate active refolding by the Hsp70 chaperone system [54,55]. The ability of Hsp27 to readily dissociate from complexes may assist in this process. Additionally, compared with αB-c, Hsp27 subunits have weaker intermolecular affinities, evidenced by a higher propensity for Hsp27 oligomers to dissociate into smaller species upon dilution [2,10,24,32,56] and an increased rate of subunit exchange between homo-oligomers in solution [57,58], processes which have been thought to increase Hsp27 chaperone activity [28,29,59]. In this work, it was necessary to use an Hsp27 mutant that does not have a cysteine within the dimer interface of the α-crystallin domain (this facilitated site-specific labelling with the fluorophore at the cysteine residue added at the extreme C-terminus), which further weakens the affinity between subunits [59]. The weaker affinity between Hsp27 subunits compared to αB-c may contribute to the apparent mechanistic differences observed here between Hsp27 and αB-c with regard to their capacity to inhibit the aggregation of FLUC and rhodanese since the increased availability of small, chaperone-active Hsp27 subunits may account for its increased efficacy in inhibiting the aggregation of client proteins compared to αB-c.

### Conclusions

By using a combination of bulk biochemical and single-molecule techniques, we have directly observed, quantified and compared the complexes formed by two human sHsps, αB-c and Hsp27, with various model client proteins. The results of this work indicate there are key mechanistic similarities and differences in how these sHsps interact with clients. Both sHsps rapidly form complexes with client proteins that expose significant amounts of hydrophobicity during their aggregation, with both sHsps sharing a mechanism whereby small sHsp subunits initially bind to client proteins, with additional sHsp subunits being recruited into these complexes over time. In addition, we show that Hsp27 interacts transiently with some clients to inhibit aggregation. Elucidating the mechanisms by which molecular chaperones interact with clients to inhibit aggregation is essential to understanding this highly conserved aspect of proteostasis.

## Materials and methods

### Materials

All common laboratory materials used in this work were purchased from Sigma-Aldrich (St Louis, MO, U.S.A.) unless otherwise stated. Purified and AF488-labelled SSB, used as a non-chaperone (negative) control in the single-molecule assays, was a kind gift from Dr Lisanne Spenkelink (University of Wollongong, Australia).

### Protein expression and purification

The amino acid sequences of all the proteins used in this work can be found in Supplementary Figure S9.

### Clients

FLUC^ΔCys, K141C, K491C^ (with N-terminal 6x-His tag and C-terminal AviTag; referred to throughout as FLUC) was expressed and purified as previously described [60]. CLIC1^C24^ (an isoform of CLIC1 harbouring mutations of five of the six native cysteines to alanines – C59A, C89A, C178A, C191A, C223A; the remaining cysteine C24 was not modified so it could be used for site-specific fluorescent labelling; this isoform is referred to as CLIC throughout) was expressed and purified as previously described [38]. To express and purify rhodanese, *Escherichia coli* (*E. coli*) BL21 (DE3) cells co-transformed with plasmids encoding biotin ligase (BirA) and SUMO-tagged Avi-rhodanese^K135C, K174C^ (an isoform with cysteine residues replacing the lysines at positions 135 and 174 for site-specific labelling, as well as an N-terminal 6x-His tag and C-terminal AviTag, referred to as rhodanese throughout this work) were used to inoculate a starter culture consisting of LB media supplemented with kanamycin (50 μg/ml) and chloramphenicol (10 μg/ml) antibiotics and grown overnight at 37°C. The starter culture was used to inoculate expression cultures containing LB media supplemented only with kanamycin (50 μg/ml), and the cultures were incubated at 37°C until an OD_600_ of ~0.4 was reached. Expression cultures were then further incubated at 18°C until an OD_600_ of ~0.6–0.8 was reached. To promote the *in vivo* biotinylation of the Avi-tagged recombinant proteins, the media were supplemented with D-biotin (50 μM final concentration) prepared in 10 mM bicine buffer (pH 8.3) prior to protein induction. The expression of recombinant protein was induced by the addition of IPTG (0.1 mM), and cultures were incubated on an orbital shaker at 130 rpm overnight (~20 hour) at 18°C. The cells were then harvested by centrifugation at 5,000×g for 10 min at 4°C and the pellet was stored at −20°C until the recombinant protein was extracted.

Recombinant SUMO-tagged rhodanese was extracted from the bacterial pellet via resuspension in 50 mM Tris-HCl (pH 8.0) supplemented with 300 mM NaCl, 5 mM imidazole, 10% (v/v) glycerol and 20 mM sodium thiosulfate (SUMO IMAC buffer A) that also contained lysozyme (0.5 mg/ml) and EDTA-free cocktail protease inhibitor. The resuspended pellet was then incubated at 4°C for 20 min. The lysates were subjected to probe sonication for 3 min (10 s on/20 s off) at 45% power. The cell debris was then pelleted twice at 24,000×g for 20 min at 4°C and the soluble bacterial lysate passed through a 0.45-μm filter to remove particulates prior to subsequent purification.

The lysate containing SUMO-tagged recombinant rhodanese was first subjected to IMAC chromatography. The bacterial lysate was loaded onto a 5-ml His-Trap Sephadex column pre-equilibrated in SUMO IMAC buffer A. The bound protein was then eluted by the addition of the SUMO IMAC buffer A supplemented with 500 mM imidazole (SUMO IMAC buffer B). The fractions containing rhodanese were pooled and dialysed in the presence of Ulp1 (4 μg/mg of recombinant protein) overnight at 4°C against SUMO IMAC buffer A that did not contain imidazole (SUMO IMAC buffer C). Cleaved recombinant rhodanese was then further purified by passing the dialysed solution over the same His-Trap Sephadex IMAC column equilibrated with SUMO IMAC buffer C and purified as described above; however, this time the recombinant protein did not bind to the column and so was collected in flow through fractions. The presence of recombinant protein in the eluate fractions was confirmed by SDS-PAGE.

Fractions containing recombinant protein were pooled and dialysed into 10 mM Tris (pH 9.0) supplemented with 0.5 mM EDTA and 10% (v/v) glycerol (IEX buffer A) for further purification by ion-exchange (IEX) chromatography. Dialysed protein (5 ml) was loaded onto a MonoQ 5/50 column pre-equilibrated in IEX buffer A and non-bound proteins eluted from the column with IEX buffer A. Recombinant rhodanese was eluted from the column using a linear salt gradient (0–500 mM NaCl) over 20 column volumes. Eluate fractions were analysed by SDS-PAGE for purity, and those fractions containing recombinant rhodanese were pooled and dialysed into storage buffer (50 mM Tris, pH 7.5, 10 mM MgCl_2_, 5 mM KCl, 10% [v/v] glycerol) overnight at 4°C. Dialysed protein was concentrated, snap frozen in liquid nitrogen and stored at –80°C until required.

## Molecular chaperones

Plasmids encoding for αB-c_C176_ (i.e. an αB-c isoform with an additional cysteine residue, C176, at the extreme C-terminus that could be used for site-specific labelling of the protein, referred to throughout as αB-c) were transformed into chemically competent *E. coli* BL21(DE3) cells, and protein expression and purification was performed as previously described [61]. To generate the Hsp27 to be used in this work, *E. coli* cells transformed with plasmid encoding for a cysteine mutant of Hsp27 (i.e. Hsp27_C137S,C207_ in which the cysteine in the α-crystallin domain, C137, was replaced with a serine and an additional cysteine was added at the extreme C-terminus, C207, that could be used for site-specific labelling of the protein, referred to throughout as Hsp27) were inoculated in LB containing ampicillin (100 μg/ml, 150 ml) and incubated overnight (37°C) with shaking (200 rpm). This culture was added to expression cultures of LB media containing ampicillin (100 μg/ml) and incubated with shaking until it reached an OD_600_ of 0.6–0.8. IPTG was added to a final concentration of 0.25 mM and incubated at 37°C for further 4 hours with constant agitation. Cells were harvested for extraction by centrifugation (5,000×g, 10 min, 4°C). The cell pellet was stored at –20°C until extraction.

In order to extract recombinant Hsp27, the pellet was resuspended in cell lysis buffer (100 mM Tris–HCl, 10 mM EDTA) containing EDTA-free protease inhibitor cocktail and lysozyme, before incubating on ice for 25 min. The cells were subjected to probe sonication (45% amplitude, 5 s on/10 s off for 3 min) and then centrifuged (24,400×g, 20 min, 4°C). The supernatant was removed and sterile filtered prior to anion-exchange chromatography. The recombinant Hsp27 was purified from contaminating bacterial proteins using anion-exchange and SEC. A diethylaminoethyl (DEAE) FF 16/10 anion exchange column (GE Healthcare Lifesciences) equilibrated with DEAE buffer A (20 mM Tris-HCl, pH 8.5, 1 mM EDTA, 0.02% (w/v) NaN_3_) was used as the first step in purification procedures. The cell lysate was loaded onto the column and bound protein eluted using a linear gradient of NaCl from 0 to 200 mM over 8 column volumes by the addition of DEAE buffer B (20 mM Tris-HCl, pH 8.5, 1 mM EDTA, 0.02% (w/v) NaN_3_, 2 M NaCl,). The eluate was monitored for protein absorbance at 280 nm and fractions collected from the column analysed by 12% (w/v) SDS-PAGE. Fractions containing Hsp27 were pooled and concentrated prior to loading onto a Sephacryl s300 size-exclusion column (GE Healthcare Lifesciences) previously equilibrated with 50 mM phosphate buffer (pH 7.4, PB). Again, fractions containing the purified Hsp27 were identified by SDS-PAGE, pooled and concentrated. Aliquots were snap frozen in liquid nitrogen prior to storage at −80°C.

### *In vitro* aggregation assays

The ability of the sHsp forms to be used in the single-molecule assays to prevent the amorphous aggregation of each client protein was assessed by measuring light scatter using *in vitro* aggregation assays. The clients CLIC (30 µM), FLUC (4 µM) and rhodanese (4 µM) were incubated in 50 mM phosphate buffer (pH 7.4) and 10 mM diothiothreitol in the absence or presence of varying molar ratios (1:1, 1:2, 1:4, client:sHsp) of αB-c and Hsp27 or the non-chaperone control protein, ovalbumin (1:4, client:ovalbumin). Samples were prepared in duplicate in a clear-bottom 384-well microplate (Greiner Bio-One, Austria) to a final volume of 50 µl per well and incubated at 42°C without shaking in FLUOstar Optima plate reader (BMG Lab Technologies, Melbourne, Australia) to cause the amorphous aggregation of the client protein. The light scatter within each sample was continually monitored at 360 nm over the course of the incubation. For each experiment, technical replicates were averaged and used to calculate the relative ability of sHsps to inhibit client aggregation. This was reported as the percentage protection afforded by each sHsp, calculated using equation 1:


(1) $$\%Protection=\frac{\Delta -\Delta sHsp}{\Delta }\times 100$$

**Equation 1. The equation used to calculate the percentage protection afforded by the sHsps**.

△sHsp and △ represent the maximum change in light scatter of the client in the presence and absence (respectively) of sHsp (or control protein) over the course of the incubation. The % protection is reported as the mean ± S.D of three independent replicates.

### Bis-ANS assay

Changes in hydrophobicity upon thermal denaturation of client proteins were monitored by 4,4′-dianilino-1,1′-binaphthyl-5,5′-disulfonic acid (bis-ANS). CLIC, FLUC or rhodanese (200 nM) was incubated with bis-ANS (20 µM) in 50 mM sodium phosphate buffer (pH 7.4) at room temperature for 10 min. These protein solutions were then dispensed in triplicate into a clear-bottom 384-well microplate (Greiner Bio-One, Austria) to a final volume of 50 µl per well and incubated at 37°C without shaking in FLUOstar Optima plate reader (BMG Lab Technologies, Melbourne, Australia) for 10 hours to promote denaturation of the client, and the bis-ANS fluorescence was measured every 5 min (ex: 355 nm, em: 480 nm). Each emission value was corrected by subtracting a sodium phosphate buffer/bis-ANS blank. The change in bis-ANS fluorescence over time was calculated for each client protein by subtracting the bis-ANS fluorescence value at the first reading, from all corresponding readings after that. The rate of change in bis-ANS fluorescence was calculated by fitting the data to a one-phase association model using GraphPad Prism 9.0 (GraphPad Software Inc.).

### Fluorescent labelling of proteins

Alexa Fluor 488-maleimide (AF488) fluorophore and Alexa Fluor 647-maleimide (AF647) (ThermoFisher Scientific, Waltham, Massachusetts, United States) were used to label the sHsps (Hsp27 and αB-c, or the control protein SSB) and clients (CLIC, FLUC and rhodanese), respectively (Supplementary Figure S9). To do so, 5 mM tris(2-carboxyethyl)phosphine (TCEP) was added to protein (1 mg/ml) to reduce any existing disulphide bonds. Ground ammonium sulfate was dissolved in the protein solution to 40% (w/v), and the sample rotated for 1 hour in order to precipitate the protein from solution.

Buffer A (100 mM sodium phosphate, pH 7.4, 200 mM NaCl, 1 mM EDTA, 70% [w/v] ammonium sulfate) and buffer B (100 mM sodium phosphate, pH 7.4, 200 mM NaCl, 1 mM EDTA) were degassed using the Shlenk line technique [62]. Briefly, each buffer was sealed with a rubber plug, and a syringe bound to an argon-filled balloon was inserted into the solution. A ‘purge’ syringe was used to degas the buffer for 1 hour (buffer A) or 30 min (buffer B).

Following protein precipitation by ammonium sulfate, the solution was centrifuged (20,000×g, 15 min, 4°C) to pellet the precipitated protein, and the pellet was washed by the addition of buffer A (200 μl) followed by centrifugation (20,000×g, 15 min, 4°C). The pellet was then resuspended in buffer B (95 μL) and a five-fold molar excess of fluorophore in DMSO, added such that the final concentration of DMSO was 5% (v/v). The reaction vessel was covered with foil to block light and mixed via rotation for 2 hours at room temperature or, in the case of Hsp27, 37°C overnight.

The sample was loaded onto a Zeba Spin Desalting Column (7K MWCO, 0.5 ml, ThermoFisher Scientific) and pre-equilibrated with 50 mM PB (pH 7.4) as per manufacturer instructions. This was repeated using as many spin columns as required to remove free dye molecules. The protein concentration and degree of labelling (DOL) were determined using the Nanodrop 2000/2000c (ThermoFisher Scientific, Waltham, Massachusetts, United States) and Equations 2 and 3. The labelling yield and molar extinction co-efficient of each protein can be found in Supplementary Table S1 . The amount of free dye within the solution was assessed by SDS-PAGE analysis.


[Protein]=(A280−(Maxdyeabsorbance∗C.F.))/(ϵprotein)

**Equation 2. The equation used to calculate the molar concentration of labelled protein**.

C.F. refers to the correction factor, which was used to account for the spectral crossover between the fluorophore emission wavelength and the protein at 280 nm. C.F (AF488) = 0.11, C.F (AF647) = 0.03. A280 is the absorbance of the protein at 280 nm, and ɛ is the molar extinction co-efficient of the protein.


$$DOL=(Maxdyeabsorbance)/(\epsilon_{dye}\times [protein(M)])\times 100$$

**Equation 3. The equation used to calculate the labelling efficiency of maleimide-labelled proteins**.

ε_dye_ refers to the extinction co-efficient of the fluorophore [εdye (AF488) = 72,000 M^-1^ cm^-1^ , εdye (AF647) = 83,000 M^-1^ cm^-1^].

### Single-molecule TIRF sample preparation

To confirm the presence and composition of complexes formed between clients and sHsps, single-molecule TIRF experiments were performed. AF647-labelled CLIC, FLUC or rhodanese (2 μM) was incubated with AF488-labelled Hsp27, αB-c or, as a non-chaperone control, SSB (4 μM) in 50 mM phosphate buffer (pH 7.4) for 7 hours at 42°C. Aliquots were taken from the reaction at 0, 0.25, 0.5, 1, 4, and 7 hours and cross-linked using bis(sulfosuccinimidyl)suberate (BS^3^) to ensure that the dilution required for single-molecule TIRF imaging did not result in dissociation of any complexes formed.

In order to amine-amine crosslink complexes at each time point, BS^3^ was dissolved in water to 12.5 mM and immediately added to the protein samples to a final concentration of 250 μM and incubated at room temperature for 30 min. Following incubation, any free crosslinking reagent was quenched via addition of 20 mM Tris hydrochloride (Tris-HCL, pH 7.5) and incubated at room temperature for 15 min before storing at 4°C until imaging.

For imaging, each timepoint sample was diluted to between 1–20 nM into imaging buffer and loaded into flow cells for imaging using TIRF microscopy.

### Calculating the number of fluorophores conjugated per client molecule

To account for the possibility of multiple fluorophores becoming conjugated to the FLUC and rhodanese molecules (due to the two available cysteine residues), the number of fluorophores per labelled FLUC/rhodanese monomer was quantified via single-molecule photobleaching. Rhodanese or FLUC was diluted to 500 pM into 6 mM 6-hydroxy-2,5,7,8-tetramethylchroman-2-carboxylic acid buffer (imaging buffer) containing oxygen scavenger system (OSS) (a combination of protocatechuic acid [PCA, 2.5 mM] and protocatechuate-3,4-dioxygenase [PCD, 50 nM]) and guanidine hydrochloride (2 M) to ensure all proteins were monomeric. A sample (50 μl) was added to a poly-L-lysine-functionalised coverslip and imaged until all molecules had photobleached. The average number of fluorophores per molecule was calculated (Supplementary Table S2) as described here for all single-molecule photobleaching analysis. For all subsequent analyses which report the number of subunits within each FLUC/rhodanese molecule (both in complexes and not in complexes), the number of subunits per molecule was divided by the average fluorophore count to correct for any molecules that had multiple fluorophores attached.

### Coverslip and flow cell preparation for single-molecule TIRF microscopy

Glass coverslips (24 mm × 24 mm) were cleaned by water bath sonication for 30 min in 100% ethanol followed by 30 min in KOH (5 M). This was repeated once, followed by sonication in Milli-Q water (5 min). Clean coverslips were dried with compressed nitrogen gas.

In order to functionalise poly-L-lysine coverslips for quantification of the number of fluorophores conjugated per protein molecule, cleaned coverslips were incubated with poly-L-lysine solution (0.01%) (100 μl) in a humidity chamber to prevent drying for 30 min. The coverslips were rinsed with Milli-Q water and dried again with compressed nitrogen gas.

In order to functionalise coverslips for use within flow cells, following the cleaning of coverslips, 5% (v/v) (3-aminopropyl) triethoxysilane (98%, Alfa Aesar, USA) was added to coverslips in Milli-Q water and incubated for 15 min. The coverslips were rinsed with Milli-Q water and dried with compressed nitrogen gas. Methoxyl polyethyleneglycol(mPeg)-succinimidyl valeric acid (SVA) and mPeg-biotin-SVA in 50 mM 3-(Nmorpholino) propanesulfonic acid (MOPS) buffer was added and incubated overnight before rinsing with Milli-Q water and drying again with compressed nitrogen gas. NeutrAvidin (an analog of streptavidin) (ThermoFisher Scientific) was applied directly to the coverslip and incubated for 15 min before rinsing with Milli-Q water and drying with nitrogen gas.

Polydimethylsiloxane (PDMS) custom microfluidics devices were applied directly to the NeutrAvidin functionalised coverslip. Inlets and outlets were inserted at each channel in the PDMS flow cell using PE-20 tubing (Instech, PA, U.S.A.), which facilitated the addition of samples into each channel. Polyoxyethylenesorbitanmonolaurate (2% [w/v] Tween20) was incubated within each microfluidic channel and incubated for 30 min to block non-specific protein binding sites and washed out with imaging buffer prior to imaging. For immobilisation of complexes formed with His-tagged CLIC, the flow cell was incubated with a biotinylated anti-6X His tag antibody (cat #ab27025, Abcam, Cambridge, MA) (1 µg/ml) for 10 min and washed with imaging buffer again.

Lastly, cross-linked samples from each time point were diluted into buffer consisting of imaging buffer and an OSS. The OSS is a combination of PCA (2.5 mM) and PCD (50 nM). Samples were incubated within the flow cell for 15 min to bind the coverslip (complexes formed with biotinylated FLUC or rhodanese bound directly to the NeutrAvidin functionalised coverslip), before washing with imaging buffer again prior to image acquisition.

### TIRF microscopy instrument setup and data acquisition

All TIRF experiments were performed at room temperature (20°C) using a custom-built system around an inverted microscope (Nikon Eclipse TI). Circularly polarised lasers with constant emission at 488- or 637-nm (200 mW Sapphire, Coherent, Santa Clara, CA, U.S.A.) excited samples labelled with AF488 or AF647, respectively. Laser light was directed from a dichroic mirror (ZT405/488/561/635, Semrock, U.S.A.) onto the sample through the back-aperture of a 60 × 1.49 NA TIRF objective (CFI Apochromate TIRF Series 60 × objective lens, numerical aperture = 1.49), coated with immersion oil creating an evanescent wave onto coverslip. Emission from the fluorophores passed through the objective, onto the same dichroic mirror. The emission of separate fluorophores was then split using a T635lpxr-UF2 longpass dichroic mirror (Chroma, U.S.A.) and then passed through ET525/50 m and ET690/50 m (Chroma, U.S.A.) filters to clean up the emission signal. The final emission signal was then projected onto the electron multiplied charge coupled device (Andor iXon Life 897, Oxford Instruments, U.K) as two separate channels. Where FRET was not observed, both lasers were used simultaneously for photobleaching the fluorophores; when FRET was observed, an alternate excitation imaging scheme was used whereby the AF647-labelled proteins were excited until all molecules photobleached, followed by subsequent photobleaching of the AF488-labelled proteins. The camera was operated at −70°C with a pixel distance of 160 nm and electron multiplication gain of 200. Single-frame images of TetraSpeck™ fluorescent microspheres (ThermoFisher Scientific) excited by the 488-nm and 637-nm laser were taken prior to each experiment for subsequent emission channel alignment. For samples of interest, image stacks were recorded with 500-ms exposure time, 600–1000 images per stack, depending on the time taken for 90% of all molecules in the field of view to photobleach completely.

### TIRF data analysis

All TIRF data were first corrected for electronic offset, uneven excitation beam distribution across the field of view and misalignment of emission channels (using the fluorescent microsphere images as alignment references for accuracy). Then, using custom written ImageJ scripts [63], each fluorescent molecule was detected, and its’ pixel-wise location within the field of view extracted and compared with the corresponding emission channel for determining colocalisation between the two fluorophores; intensity vs. time trajectories were extracted for each fluorescent spot in order to perform subsequent photobleaching analysis (Supplementary Figure S10). To account for instances wherein the pixel-wise coordinate of a fluorescently-labelled chaperone matched that of a client protein in the corresponding emission channel by chance (i.e. non-specific binding), an additional image-processing step was performed for each flow cell in each experiment. The emission channel identifying chaperones was horizontally and vertically rotated 180°, and colocalisation analysis was performed on these data. The proportion of client molecules which were colocalised in these ‘flipped’ channels (i.e*.* chance co-localisation) was subtracted from the corresponding colocalisation data for that experiment.

Photobleaching analysis was performed using a custom-written analysis package to determine the size of every molecule within each treatment and time point (Supplementary Figure S11). Briefly, all photobleaching trajectories were classified based on the shape of the data using a residual neural network (ResNet) [64] built using TensorFlow [65] and trained using example photobleaching trajectories extracted from the bleaching of AF647- and AF488-labelled proteins and manually classified prior to training. This model was used to select for trajectories that contained discrete photobleaching steps and as a quality control step to filter out molecules not suitable for further analysis. Photobleaching step sizes (in fluorescence intensity A.U.) were quantified via change point identification within the trajectories by fitting them to a Bayesian Offline model using the sdt [66] python package. The median size of all final photobleaching steps in all well-defined trajectories were calculated and used in subsequent analysis steps as the size of one photobleaching step (I_step_). The initial intensity of all suitable trajectories was extracted (the average of the first 20 intensity values of each trajectory) and defined as I_initial_. For each molecule, the number of subunits within the fluorescent spot was calculated by I_initial_/I_step_ as described previously [38]. Throughout the manuscript, the ‘size’ of the complexes refers to the number of subunits.

All further filtering and colocalisation analysis was performed using custom-written Python scripts. Plotting used seaborn [67] and matplotlib [68] data visualisation libraries

### Statistics

Statistical analysis of the aggregation assays was performed using GraphPad Prism (9.0). Single-molecule data were analysed using the SciPy [69] or Scikit [70] statistical analysis packages in Python where applicable. Kruskal–Wallis tests with Dunn’s procedure were performed for comparisons between all stoichiometric distributions. A two-way ANOVA with a Tukey’s HSD was performed to compare molecule size data between colocalised and non-colocalised molecules over time. All other specific tests are indicated in the appropriate figure legends. A *P* value of less than 0.05 was considered statistically significant, and code used to perform statistical analysis can be found in the code repository.

## Supplementary material

Online supplementary material 1

## Data Availability

All scripts used in the analysis workflow described in this work, as well as those that produced the figures and statistical analyses can be found at https://doi.org/10.5281/zenodo.10616736 [73]. Example unprocessed data have been provided to explore the analysis workflow described here. Additionally, the data to produce the figures and the statistics have also been provided and both can be found at https://doi.org/10.5281/zenodo.10602864 [74].

## Abbreviations

CLIC: chloride intracellular channel 1
FLUC: firefly luciferase
TIRF: total internal reflection fluorescence
sHsps: small heat shock proteins
