# Supplementary material for: Single-molecule observations of human small heat shock proteins in complex with aggregation-prone client proteins
Source: Biochem J. 2025 Apr 25;482(9):413–32. doi: 10.1042/BCJ20240473 (PMC12203938; doi:10.1042/BCJ20240473)
Supplement: Online supplementary material 1 [file BCJ-482-09-BCJ20240473-s001.pdf]

Single-molecule observations of human small heat shock proteins in complex with aggregation-prone client proteins

*Lauren Rice[1], Nicholas Marzano[1], Dezerae Cox[1,2], Antoine van Oijen[1], Heath Ecroyd [*1]*

[1] Molecular Horizons and School of Chemistry and Molecular Bioscience, University of Wollongong, Wollongong, NSW, Australia

[2] Department of Chemistry, University of Cambridge, Cambridge, CB2 1EW, United Kingdom

* Correspondence to Heath Ecroyd (heathe@uow.edu.au); Ph +61 2 4221 3443

Supplementary Material

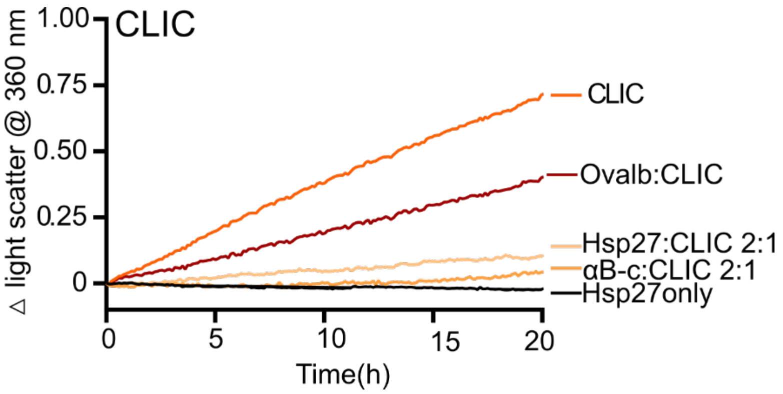

Supplementary figure 1. Heat-induced amorphous aggregation of CLIC. Example light scatter traces from the heat-induced aggregation of CLIC. Recombinant CLIC at 30 µM was incubated at 42°C for 20 h in the presence or absence of a 2:1 molar ratio (sHsp:client) of either αB-c or Hsp27, or the control protein ovalbumin (ovalb), and the change in light scatter at 360 nm over time monitored. Data shown are the normalised change in light scatter.

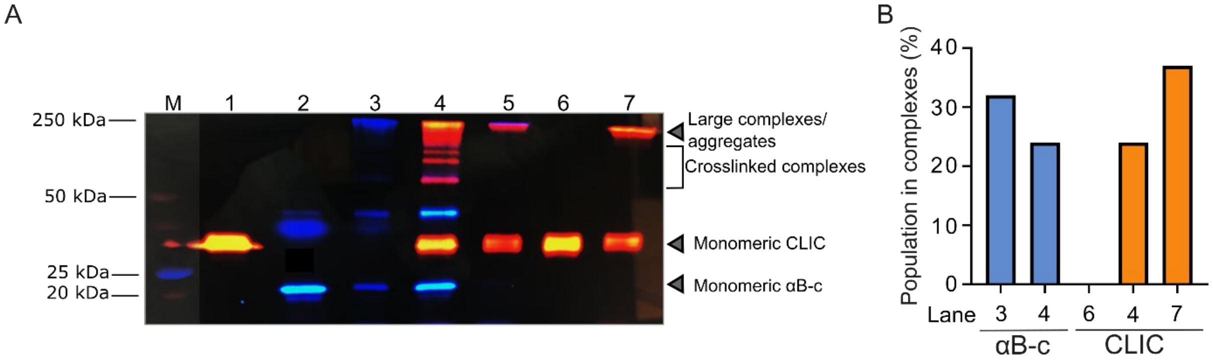

Supplementary figure 2. Chemical crosslinking maintains sHsp oligomer size and prevents sHsp:client dissociation. (A) Samples containing AF488-labelled αB-c-(2 µM) or AF647-labelled CLIC (1 µM) were incubated in the absence and presence of one another (4 h, 42°C). Samples were chemically crosslinked using 20 x molar excess of BS^3. Samples were subjected to SDS-PAGE and imaged for the corresponding fluorophore to confirm crosslinking, and compared to non-crosslinked controls. Gel shows non-crosslinked CLIC (1), non-crosslinked αB-c (2), crosslinked αB-c (3), αB-c and CLIC incubated together and crosslinked (4), crosslinked αB-c, incubated with CLIC and then crosslinked following incubation (5), crosslinked, unheated CLIC (6), incubated and crosslinked CLIC (7). (B) The percentage of αB-c (blue) and CLIC (orange) which have formed oligomers and become successfully crosslinked. In lanes 1-4, 6 and 7, the intensity of the bands which correspond to monomeric CLIC or αB-c was quantified using densitometry. The percentage of the population which was within crosslinked complexes was calculated by determining the percentage decrease in intensity of the monomeric band when the sample was crosslinked compared to the corresponding non-crosslinked sample.

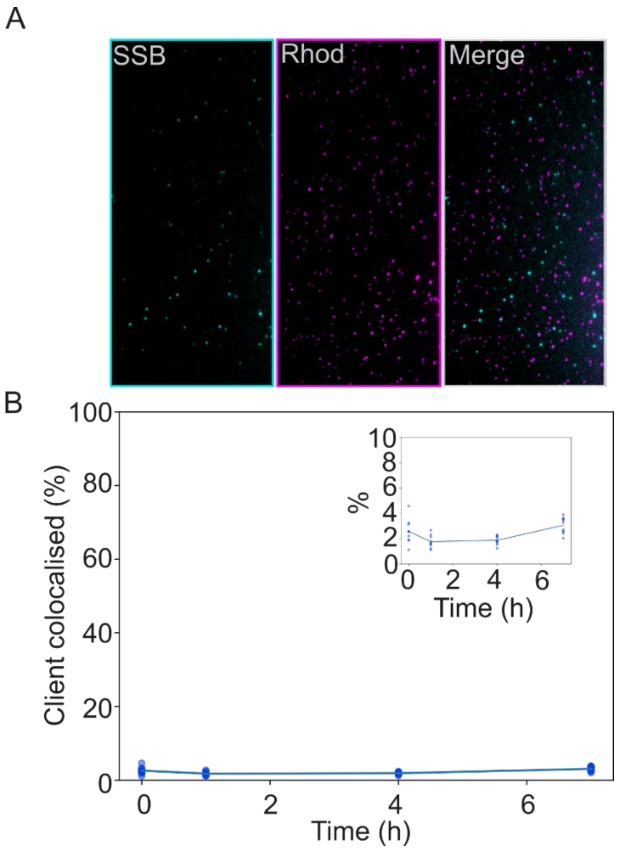

Supplementary figure 3. Minimal co-localisation occurs between a non-chaperone protein and an aggregation-prone client protein. Single-stranded binding protein (SSB – a non-chaperone control) labelled with AF647 and the client protein rhodanese (rhod) labelled with AF488 were incubated (42°C for 7 h) at a 2:1 molar ratio (SSB:client); aliquots were taken at time points throughout the incubation. Following incubation, samples were immediately crosslinked, diluted, and incubated in flow cells for 15 min before imaging using TIRF microscopy. (A) Representative TIRF microscopy image showing AF488-labelled SSB (cyan, left panel) and AF647-labelled rhodanese (magenta, middle panel) and the two channels overlaid to identify any colocalised molecules (merge, right panel). The amount of co-localisation detected was less than 3% of the total molecules detected. (B) The percentage (%) of rhodanese-AF647 colocalised with SSB-AF488 at each time point, determined from 11 images taken over two separate flow cells. Data is shown as the mean ± S.D. The inset shows the same data, with a shortened y-axis to visualise the spread of the data points.

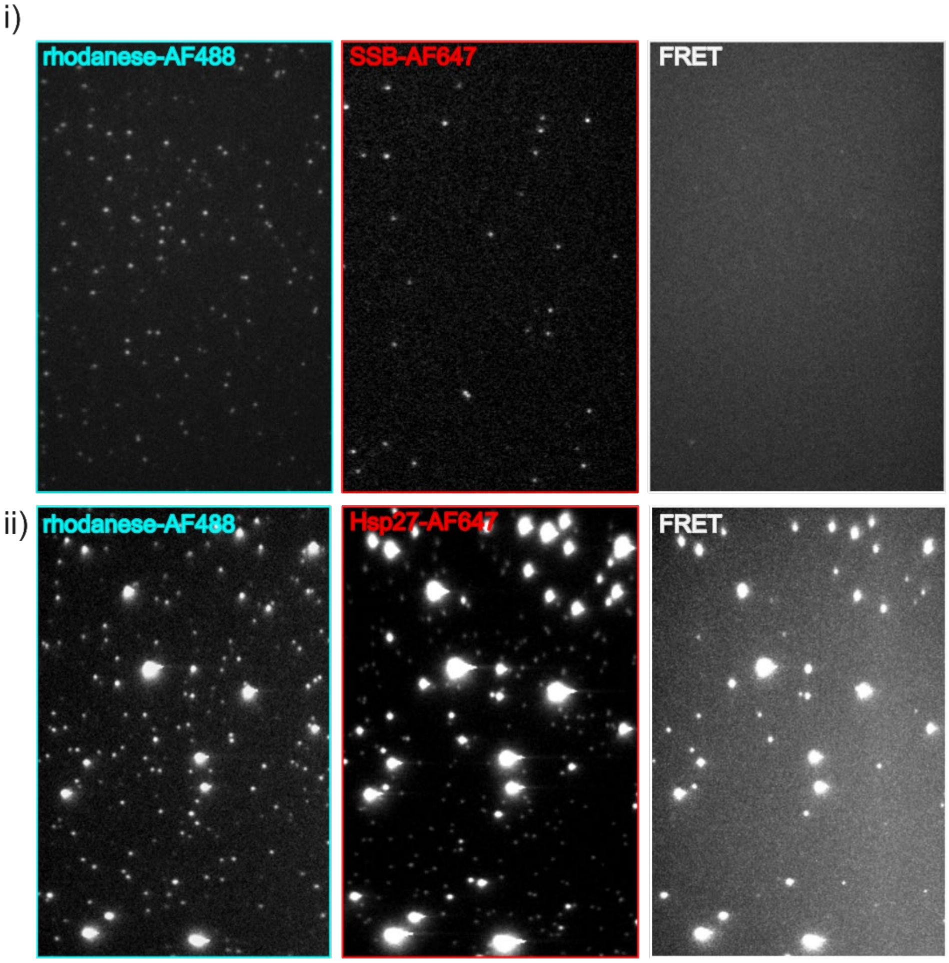

Supplementary figure 4. sHsp:client complexes form complexes which exhibit FRET. Either i) SSB as a non-chaperone control protein, or ii) Hsp27, labelled with AF647, were incubated at a 2:1 molar ratio (SSB/Hsp27:client) (42°C for 1 h) with the AF488-labelled client protein rhodanese. Following incubation, samples were immediately crosslinked, diluted, and incubated in flow cells for 15 min before imaging using TIRF microscopy. Images show rhodanese in the 488 nm emission channel taken during 488 nm laser excitation; SSB or Hsp27 signal in the 647 nm emission channel as a result of 637 nm laser excitation; and the FRET signal in the acceptor (647 nm) emission channel as a result of 488 nm (donor) excitation only.

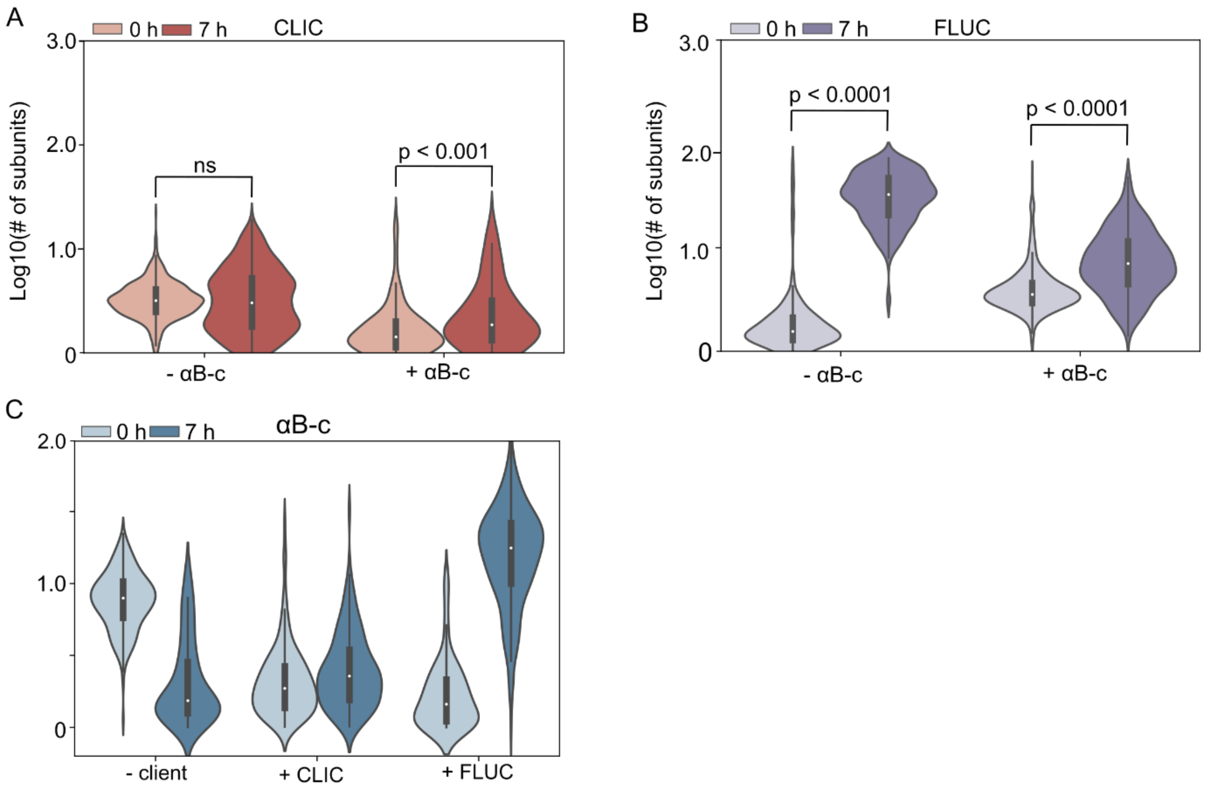

Supplementary figure 5. αB-c inhibits aggregation of CLIC- or FLUC- AF647 and the increase in size of αB-c molecules is client protein-dependent. CLIC- or FLUC-AF647 were incubated (42°C for up to 7 h) in the absence or presence αB-c (2:1 molar ratio); aliquots were taken at the start (0 h) and end (7 h) of the incubation. Following incubation, samples were crosslinked, diluted, and incubated in flow cells for 15 min before imaging using TIRF microscopy. (**A-C**) Violin plots show the distributions of all (colocalised and non-colocalised) molecule sizes (log_{10} number of subunits/molecule) prior to and after incubation of (A) CLIC-AF647 and (B) FLUC-AF647 with ('+αB-c') or without ('-αB-c') αB-c-AF488; or, (C) αB-c-AF488 alone ('-client') and in the presence of CLIC–AF647('+ CLIC') or FLUC-AF647 ('FLUC'). Results include measurements from three independent experiments and, where marked, statistical comparison between distributions was performed via Kruskal-Wallis test for multiple comparisons with Dunn's procedure (p values indicated).

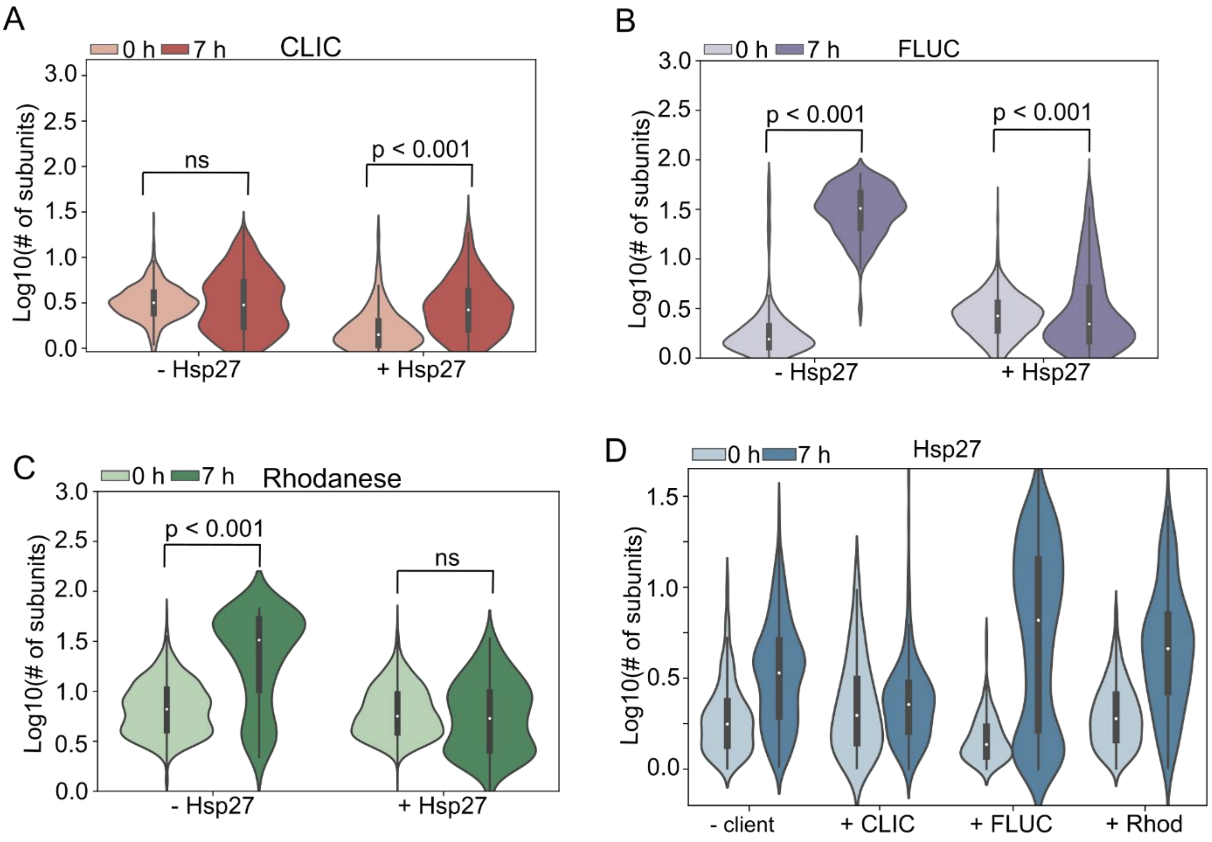

Supplementary figure 6. Hsp27 inhibits aggregation of CLIC-, FLUC- and rhodanese-AF647, and Hsp27 molecule size is client-protein dependent. CLIC-, FLUC- and rhodanese-AF647 were incubated (42°C for 7 h) in the presence (2:1 molar ratio) and absence of one another; aliquots were taken at the start (0 h) and end (7 h) of the incubation. Following incubation, samples were crosslinked, diluted, and incubated in flow cells for 15 min before imaging using TIRF microscopy. (**A-D**) Violin plots show the distributions of all (colocalised and non-colocalised) molecule sizes (log_{10} number of subunits/molecule) prior to and after incubation of (A) CLIC-AF647, (B) FLUC-AF647, and (C) rhodanese-AF647 with ('+sHsp') or without ('-sHsp') Hsp27-AF488; or, (C) Hsp27-AF488 alone ('-client') and in the presence of CLIC-AF647 ('+ CLIC'), FLUC-AF647 ('FLUC'), or rhodanese-AF647 ('Rhodanese'). Results include measurements from three independent experiments and, where marked, statistical comparison between distributions was performed via Kruskal-Wallis test for multiple comparisons with Dunn's procedure.

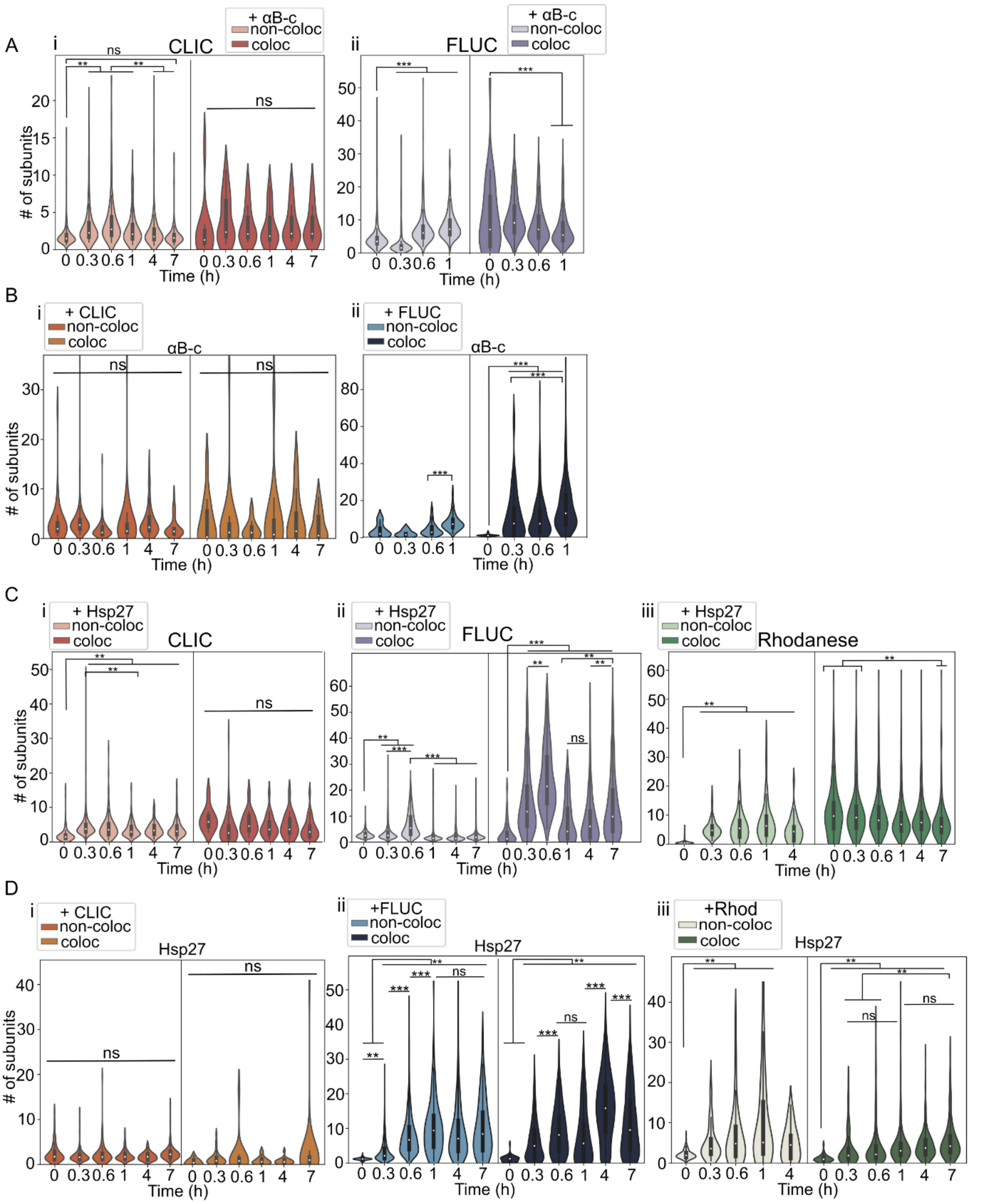

Supplementary figure 7. sHsps maintain client proteins in small oligomeric states. AF488-labelled sHsps (αB-c or Hsp27) were incubated with the AF647-labelled client proteins (CLIC, FLUC or rhodanese) (42°C for 7 h, 2:1 molar ratio) and aliquots were taken throughout the incubation. Samples were immediately crosslinked, diluted, and incubated in flow cells for 15 min before imaging using TIRF microscopy. The molecule size (# of subunits/molecule) was calculated for all molecules, and the data was filtered for the **(A, C)** client and **(B, D)** sHsp molecules that were colocalised with one another (i.e., in complexes) and those that were not colocalised (i.e., not in complexes). (A,C) Violin plots showing the distribution of the number of subunits per molecule of both colocalised ('Coloc') and non-colocalised ('Non-coloc')

molecules at each time point for the clients CLIC (i, red), FLUC (ii, purple) and/or rhodanese (iii, green) incubated with (B) αB-c or (D) Hsp27. The size of the sHsps in these treatments are shown in the corresponding plots for (B) αB-c and (D) Hsp27. The data shown contains the number of subunits from all molecules in three independent experiments. A two-way ANOVA was performed for all treatments, and, where relevant, statistical differences are marked (** = $p < 0.005$, *** = $p < 0.0005$, ns indicates no significant difference. *The median and error values presented in Figure 4 (main text) are calculated from all molecules depicted here as distributions.*

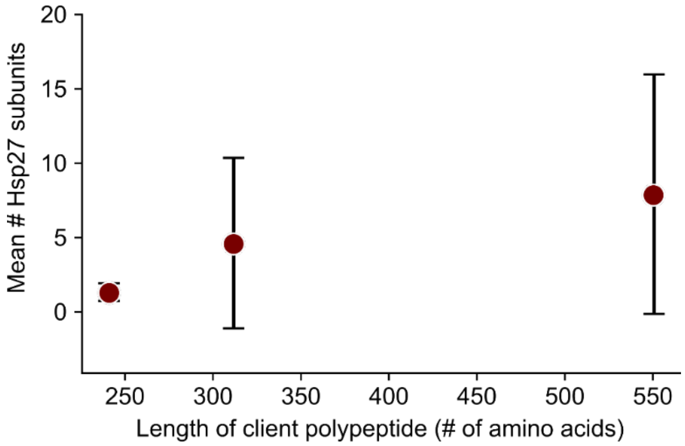

Supplementary figure 8. The number of Hsp27 subunits in complexes with client proteins in relation to the length of the client protein. AF488-labelled Hsp27 was incubated with the AF647-labelled client proteins (CLIC, FLUC or rhodanese) (42°C, 2:1 molar ratio) and aliquots were taken throughout the incubation. Samples were immediately crosslinked, diluted, and incubated in flow cells for 15 min before imaging using TIRF microscopy. The number (#) of subunits/molecule was calculated for all molecules and the data was filtered for only the Hsp27 molecules colocalised with a client protein at 1 hour of incubation. The data shown are the mean (+/- standard deviation) number of Hsp27 subunits in complexes with CLIC, rhodanese or FLUC molecules (241, 324, and 550 amino acids, respectively), from three independent experiments.

A

αB-crystallin

MDIAIHHPWIRRPFFPFHSPSRLFDQFFGEHLLESDLFPTSTSLSPFYLRPPSFLRAPSW FDTGLSEMRLEKDRF
SVNLDVKHFSPEELKVKVLGDVIEVHGKHEERQDEHGFISREFHRKYRIPADVDPLTITSSLSSDGVLTVNGPRK
QVSGPERTIPITREEKPAVTAAPKKC

B

Hsp27

MTERRVPFSLLRGPSWDPFRDWYPHSRLFDQAFGLPRLPEEWSQWLGGSSWPGYVRPLPP AAIESPAVAAPAYSR
ALSRQLSSGVSEIRHTADRWRVSLDVNHFAPDELTV KTKDGVVEITGKHEERQDEHGYISRSFTRKYTLPPGVDP
TQVSSSLSPEGTLTVEAPMPKLATQSNEITIPVTFESRAQLGGPEAAKSDETAAKC

C

Chloride intracellular channel protein 1 (CLIC)

MAEEQPQVELFVKAGSDGAKIGNCPFSQRLFMVLWLKGVTFNVTTVDTKRRTETVQKLAPGGQLPFLLYGTEVHT
DTNKIEEFLEAVLAPPRYPKLAALNPESNTAGLDIFAKFSAYIKNSNPALNDNLEKGLLKALKVLDNYLTSPLPE
EVDETSAEDEGVSQRKFLDGNELTLADANLLPKLHIVQVVAKKYRGFTIPEAFRGVHRYLSNAYAREEFASTAPD
DEEIELAYEQVAKALK

D

Firefly Luciferase (FLUC)

MEDAKNIKKGPAPFYPLEDGTAGEQLHKAMKRYALVPGTIAFTDAHIEVNITYAEYFEMSVRLAEAMKRYGLNTN
HRIVVSSENSLQFFMPVLGALFIGVAVAPANDIYNERELLNSMNISQPTVVFVSKKGLQKILNVQ CKLPIIQKII
IMDSKTDYQGFQSMYTFVTSHLPPGFNEYDFVPESFDRDKTIALIMNSSGSTGLPKGVALPHRALAVRFSHARDP
IFGNQIAPDTAILSVVPFHHGFGMFTTLGYLISGFRVVLMYRFEEELFLRSLQDYKIQSALLVPTLFSFLAKSTL
IDKYDLSNLHEIASGGAPLSKEVGEAVAKRFHLPGIRQGYGLTETTSAILITPKGDDKPGAVGKVVPFFEAKVVD
LDTGKTLGVNQRGELSVRGPMIMSGYVNNPEATNALIDKDGWLHSGDIAYWDEDEHFFIVDRLKSLIKYKGYQVA
PAELESILLQHPNIFDAGVAGLPDDDAGELPAAVVVLEHGCTMTEKEIVDYVASQVTTAKKLRGGVVFVDEVPKG
LTGKLDARKIREILIKAKKGGKSKL

E

Rhodanese

MVHQVLYRALVSTKWLAESVRAGKVGPGLRVLDASWYSPGTREARKEYLERHVPGASFFDIEE CRDKASPYEVML
PSEAGFADYVGSLGISNDTHVVVYDGDCLGSFYAPRVWWMFRVFGHRTVSVLNGGFRNWLKEGHPVTSEPSRPEP
AIFKATLNRSLLKTYEQVLENLESKRFQLVDSRAQGRYLGTQPEPDAVGLDSGHIRGSVNMPFMNFLTE CGFEKS
PEELRAMFEAKKVDLTKPLIATCRKGVTACHIALAAYLCGKPDVAIYDGSWFEWFHRAPPETWVSQGKGGKAGSE
NLYFQSMAGLNDIFEAQKIEWHEH

Supplementary figure 9. Amino acid sequences of the proteins used in this work. The single-letter amino acid sequences for (A) αB-crystallin (αB-c), (B) Hsp27, (C) CLIC, (D) FLUC, and (E) rhodanese. Exposed (green) and buried (magenta) cysteine residues are highlighted, as an indication of which residues are available for conjugation of fluorophores to each protein using maleimide chemistry. The availability of cysteines for labelling in rhodanese is based on previous work (see Kellner et al, 2014. *PNAS*; **111**, 13355; Hillger et al, 2007. *J Fluoresc*; **17**, 759).

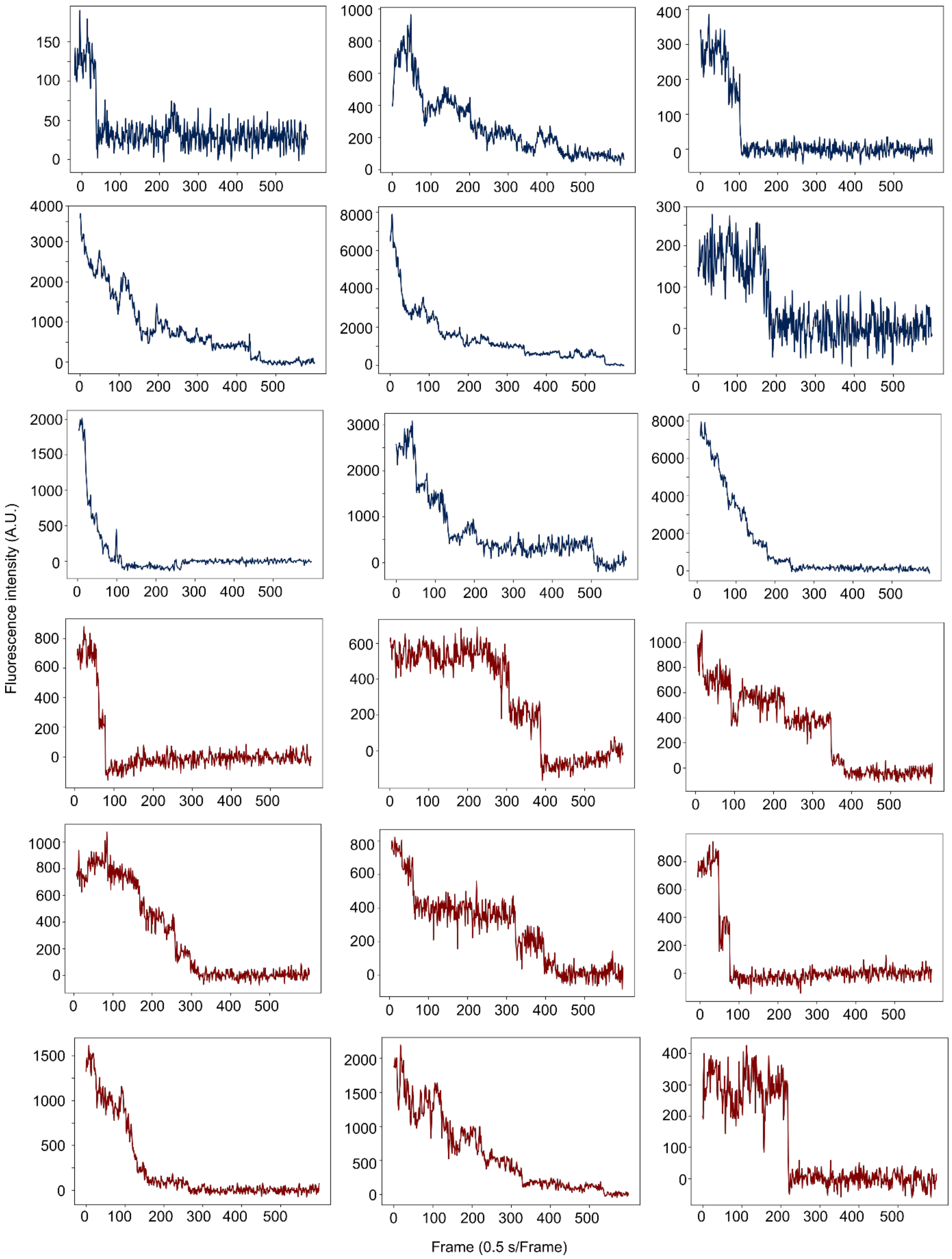

Supplementary figure 10. Example unprocessed photobleaching trajectories. (A-I) Fluorescence trajectories are from AF488-labelled sHsps (blue) or AF647-labelled client proteins from separate experiments (i.e., imaged on different days) imaged using TIRF microscopy until completely photobleached, and the fluorescence intensity of each molecule was extracted from every frame of the resulting movies. For each molecule, the intensity (fluorescence intensity (A.U.) was plotted over time (frame (0.5 s/frame)).

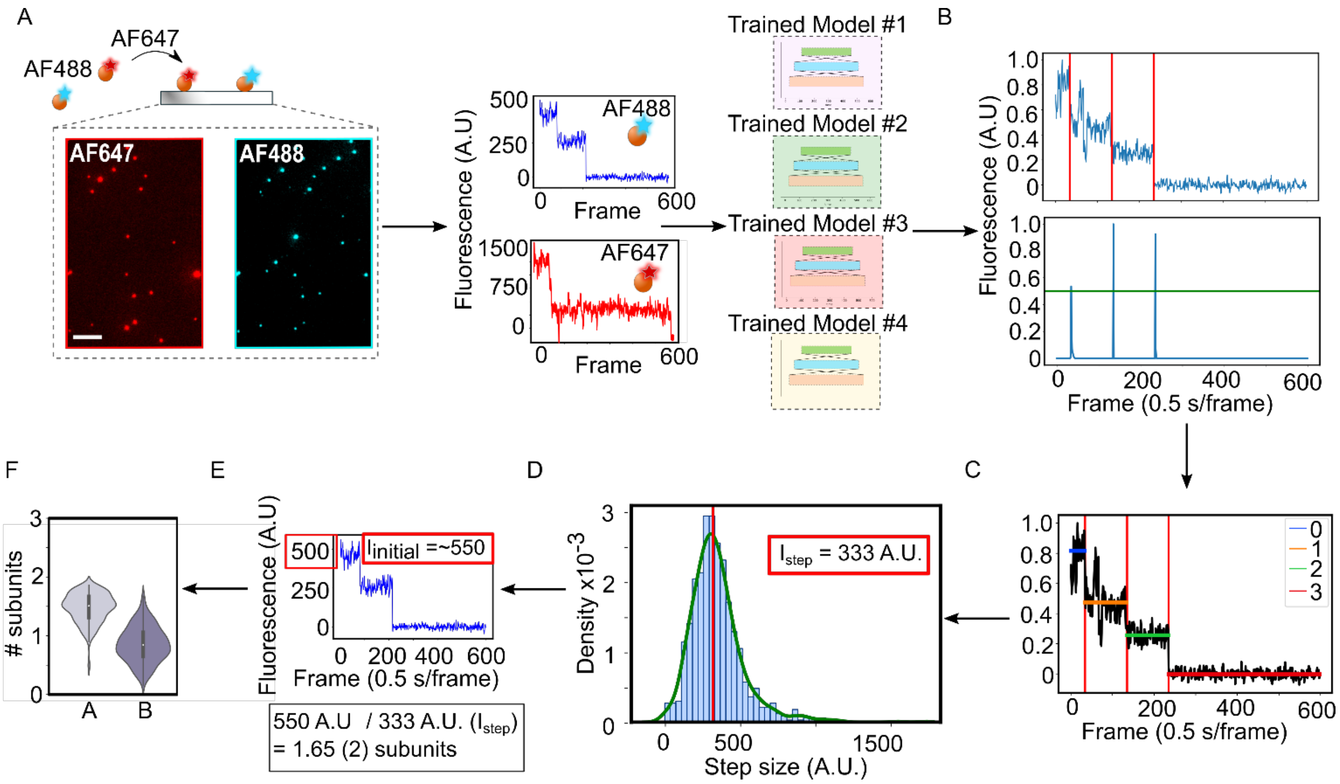

Supplementary figure 11. The Python-based analysis workflow used in this study to determine the size of molecular assemblies from TIRF-based photobleaching data. Schematic depiction of the workflow used in the automated analysis pipeline to determine the number of subunits per fluorescent molecule in a single-molecule image. (A) Biomolecules containing fluorophores of different emission wavelengths are immobilised to a coverslip surface and visualised using TIRF microscopy to obtain photobleaching trajectories. Trajectories are used to train a ResNet model to identify those with distinct photobleaching steps. (B) Bayesian offline change-point detection is performed on well-defined photobleaching trajectories; top - photobleaching trajectory (blue) and identified change-points (red), bottom - probabilities of a change occurring at each point in the corresponding trajectory (blue) and the user-defined probability threshold (green). (C) Average fluorescence at each 'step' is calculated and labelled according to the order in which it occurs. (D) The sizes of the last step from all trajectories are plotted as a distribution and the I_{step} defined as the median last step size. (E) The average starting fluorescence intensity of every trajectory is defined as $I_{initial}$ and divided by the median I_{step} to calculate the number of subunits in that molecule. (F) Violin plots show an example of the type of data generated using the analysis pipeline to determine the size of two different oligomeric proteins which were immobilised and photobleached using TIRF microscopy. Note – in this case the y-axis data are presented as $\log_{10}$ the number of subunits per molecule.

Supplementary Table 1. Summary of the labelling efficiency for each of the recombinant proteins used in this work.

Protein	Fluorophore	Degree of labelling	Molar extinction co-efficient of protein (mg $^{-1}$/mL/cm^{-1})
**αB-c**	488-C_5-mal	86%	0.83
**Hsp27**	488-C_5-mal	115%	1.77
**CLIC1**	647-C_2-mal	82%	0.55
**FLUC**	647-C_2-mal	159%	0.72
**Rhodanese**	647-C_2-mal	145%	1.81

Supplementary Table 2. The mean (± S.E) number of AF647 fluorophores conjugated to each protein monomer for proteins used in this work which contain two cysteines.

Protein	Average number of fluorophores/monomer (± S.E.)
**FLUC**	1.27 (± 0.06)
**Rhodanese**	1.07 (± 0.03)
